# MicroRNA biomarkers in leprosy: insights from the Northern Brazilian Amazon population and their implications in disease immune-physiopathology

**DOI:** 10.3389/fgene.2024.1320161

**Published:** 2024-01-25

**Authors:** Miguel Ángel Cáceres-Durán, Pablo Pinto, Leandro Magalhães, Tatiane Piedade de Souza, Angelica Gobbo, Josafá Gonçalves Barreto, Moises Batista da Silva, Patrícia Fagundes da Costa, Claudio Guedes Salgado, Ândrea Ribeiro-dos-Santos

**Affiliations:** ^1^ Laboratório de Genética Humana e Médica, Instituto de Ciências Biológicas (ICB), Universidade Federal do Pará (UFPA), Belém, Brazil; ^2^ Laboratório de Dermato-Imunologia, Instituto de Ciências Biológicas (ICB), Universidade Federal do Pará (UFPA), Marituba, Brazil; ^3^ Laboratório de Epidemiologia Espacial, Universidade Federal do Pará (UFPA), Castanhal, Brazil; ^4^ Laboratório de Patologia Geral, Universidade Federal do Pará (UFPA), Belém, Brazil

**Keywords:** leprosy, microRNA, apoptosis, autophagy, mitophagy, immune system, biomarker, Amazon

## Abstract

Leprosy, or Hansen’s Disease, is a chronic infectious disease caused by *Mycobacterium leprae* that affects millions of people worldwide. Despite persistent efforts to combat it leprosy remains a significant public health concern particularly in developing countries. The underlying pathophysiology of the disease is not yet fully understood hindering the development of effective treatment strategies. However, recent studies have shed light on the potential role of microRNAs (miRNAs), small non-coding RNA molecules that can regulate gene expression, as promising biomarkers in various disease, including leprosy. This study aimed to validate a set of nine circulating miRNAs to propose new biomarkers for early diagnosis of the disease. *Hsa-miR-16-5p*, *hsa-miR-106b-5p*, *hsa-miR-1291*, *hsa-miR-144-5p*, and *hsa-miR-20a-5p* showed significant differential expression between non-leprosy group (non-LP) and leprosy group (LP), accurately discriminating between them (AUC > 0.75). In addition, our study revealed gender-based differences in miRNA expression in LP. Notably, *hsa-miR-1291* showed higher expression in male LP, suggesting its potential as a male-specific biomarker. Similarly, *hsa-miR-16-5p* and *hsa-miR-20a-5p* displayed elevated expression in female LP, indicating their potential as female-specific biomarkers. Additionally, several studied miRNAs are involved in the dysregulation of apoptosis, autophagy, mitophagy, cell cycle, and immune system in leprosy. In conclusion, the validation of miRNA expression highlights several miRNAs as potential biomarkers for early diagnosis and provides new insights into the pathogenesis of the disease.

## 1 Introduction

Leprosy, also known as Hansen’s disease, is a neglected tropical disease of the skin and peripheral nerves caused by *Mycobacterium leprae*, presenting a strong link with the host genetic background (Fava et al., 2020). Currently, leprosy continues as a public health problem in several countries of the world. In 2022, 182 countries, areas and territories shared information on leprosy, accounting for a registered prevalence of 165.459 cases and 174.087 new cases, of which 67.657 (39%) were among females. Globally, 9.554 new cases with G2D were detected and 278 (3%) of them were among children (World Health Organization, 2022).

The *bacillus* is an obligate intracellular parasite with tropism for peripheral nervous system, and thus neural involvement is a feature of all forms of leprosy, has a slow replication, a long incubation period, and few genes controlling its metabolism. As a result, the disease progresses slowly over years or even decades, resulting in various clinical presentations and mimicking numerous other diseases; thus, treating this disease is extremely challenging. Specifically, *M. leprae* contain more than 3 million base pairs which leads to a dependence on the host’s energy production and nutritional products, resulting in parasitic life adaptation (Cole et al., 2001; Akama et al., 2009; Soares et al., 2017; Oliveira et al., 2021).

Classification of leprosy is complex and challenging, and includes clinical, histopathological, microbiological, and immunological features. The Ridley-Jopling system classifies Leprosy as a spectral disease, at one extreme of the spectrum is the polar tuberculoid form (TT), characterized by a low bacterial load, primarily cell-mediated immunity, and minor production of specific antibodies; at the other extreme of the spectrum is the polar lepromatous form (LL), characterized by a high bacterial load, increased production of anti-bodies, and lower or absent *M. leprae*-specific cell-mediated immunity. Also, there is a clinically unstable borderline spectrum between these two polar forms, border-line-tuberculoid (BT), borderline-borderline (BB), and borderline-lepromatous (BL), with BB being the least stable (Ridley and Jopling, 1966).

Leprosy is a difficult disease to treat, especially during the reactionary episodes. Current medications, such as corticosteroids and thalidomide, significantly disrupt homeostasis, resulting in difficult-to-control disorders such as obesity, diabetes, immunodeficiency, and teratogenesis, among others (Soares et al., 2017).

It is now well known that exposure to *M. leprae* alone is not enough to cause leprosy, and only a small proportion of people exposed to the *bacillus* eventually develop the disease. Indeed, it is likely that a combination of numerous variables including environmental factors, pathogen load, genetic background, socioeconomic status, time of exposure to the *bacillus*, and host immune response are related to the development of leprosy (Naaz et al., 2017; Fava et al., 2020). Currently, studies that evaluate the epigenetic role in the infection and subsequent development of the disease are beginning to emerge, and, remarkably, studies with miRNAs have advanced to several infectious diseases, including Leprosy (da Silva et al., 2022; Jorge et al., 2017; Khanizadeh et al., 2019; Salgado et al., 2018a). MicroRNAs (miRNAs) are a major class of small ncRNAs found in animals, plants, and some viruses, which regulate post-transcriptional silencing of target genes at the mRNA level (Bartel, 2004; Lu and Rothenberg, 2018). These small RNAs play a significant role in modulation of an array of physiological and pathological processes ranging from embryonic development to neoplastic progression. They mostly function by binding to complementary target sequences in mRNA and interfering with the translational machinery, thus preventing or altering the production of the protein product. Additional studies also have shown that besides repressing translation, miRNA binding to its target mRNA also triggered the recruitment and association of mRNA decay factors, leading to mRNA destabilization, degradation, and resultant de-crease in expression levels (Bhaskaran and Mohan, 2014). MiRNAs are involved in several important biological processes, such as modulation of the adaptative and innate immune system, response against pathogens, cell proliferation, cell differentiation and apoptosis (Das et al., 2016; Liu et al., 2017; Yang and Ge, 2018; Lukasik and Zielenkiewicz, 2019).

Delay in the diagnosis of leprosy can lead to the development of more advanced stages of the disease, thus increasing the chances of transmission of the infectious agent and compromising the quality of life of patients (Jorge et al., 2017). The identification of the disease remains difficult due to the limited sensitivity of traditional approaches based on bacillary counts of skin smears and histology (Sharma and Singh, 2022). Molecular techniques using PCR technology and serological tests were developed; but sensitivity and specificity were limited, because household contacts of leprosy patients, as individuals remaining without disease may present positive PCR and/or PGL-I (van Hooij et al., 2016; Jorge et al., 2017; Carvalho et al., 2018). In this sense, identification of new biomarkers is needed for the early diagnosis of leprosy, as well as to discriminate the different forms of the disease. Recently, our group presented the first leprosy miRNome from skin lesions and blood in leprosy patients (Salgado et al., 2018a), finding several miRNAs with significant differential expression. For this reason, in this work we validated these differentially expressed miRNAs in blood samples from patients and household contacts, in order to propose new leprosy biomarkers for early diagnosis of the disease.

## 2 Materials and methods

### 2.1 Biological samples

In total, 108 blood samples were collected from 49 non-consanguineous and healthy household contacts of leprosy patients, named as non-leprosy group (non-LP); and 59 leprosy patients (LP) as follows: 33 from tuberculoid-tuberculoid (TT) and borderline tuberculoid (BT) form, and 26 from lepromatous-lepromatous (LL) and borderline lepromatous (BL) form. All patient samples were obtained before starting MDT treatment at URE Dr. Marcello Candia, in Marituba, Pará, Brazil. All samples were stored in RNAlater (SIGMA R0901) and frozen at −80°C immediately after collection. This study adhered to the Declaration of Helsinki and was approved by the Ethics Committee of Institute of Health Sciences at the Federal University of Pará (26765414.0.0000.0018). Informed consent was obtained from all individual participants.

### 2.2 RNA isolation and RT-qPCR

The nine miRNAs validated were *hsa-miR-144-5p*, *hsa-miR-20a-5p*, *hsa-miR-1291*, *hsa-miR-106b-5p*, *hsa-miR-16-5p*, *hsa-miR-26b-5p*, *hsa-miR-15a-5p*, *hsa-miR-126-5p* and *hsa-let7f-5p*, all identified as dysregulated in a previous study (Salgado et al., 2018a). Total RNA was isolated from peripheral blood using TRIzol reagent (ThermoFisher, Waltham, MA, United States, catalog #15596018), according to manufacturer’s instructions. The purity and concentration of RNA samples were measured using the NanoDrop ND-1000 Spectrophotometer (Thermo Fisher Scientific). RNA samples that achieved adequate purity ratios (A260/A280 = 1.9–2.1) were used for subsequent analyses. RNA integrity was also checked on 1% agarose gels containing SYBR^®^ DNA gel stain (Invitrogen). cDNA synthesis was performed with at least 50 ng of RNA input and random hexamers using GoTaq^®^ 2 step RT-qPCR Systems (Promega, Madison, WI, United States, catalog #A6010). Quantitative real-time PCR was conducted in an *AriaMx Real-time PCR System–Agilent*, using GoTaq^®^ 2 step RT-qPCR Systems (Promega, Madison, WI, United States, catalog #A6010). The real-time PCR assays were performed in a final volume of 10 µL. Reactions consisted of 2 ng of cDNA, 250 nM of each forward and reverse primers, and 5 µL of qPCR master mix in thermal cycling conditions provided by the manufacturer. Primers utilized are listed in Supplementary Table S1. Expression levels were normalized using RNU6B and RNU24 as endogenous control. All qPCR experiments were conducted in triplicates.

### 2.3 Statistical analysis

The expression data of miRNAs for each sample were normalized to RNU6B e RNU24, using the comparative Ct method (2^−ΔΔCt^) (Schmittgen and Livak, 2008). Ct values > 35 were considered undetectable. Shapiro-Wilk test was used to verify if the normalized expression values followed a Gaussian distribution. T-test and Wilcoxon test were used to compare the miRNA expression differences in each condition and *p*-values ≤ 0.05 were considered to be statistically significant. All tests and graphs were performed in *RStudio statistical software* (*v. 4.2.2*).

### 2.4 Receiver operating characteristic (ROC)

In order to verify if miRNAs expression was able to distinguish non-LP from LP, Receiver Operating Characteristic (ROC) curves and Area Under the Curve (AUC) were calculated using the pROC package in RStudio statistical software (v. 4.2.2), and miRNAs that showed an AUC >0.75 were considered to be good potential biomarkers.

### 2.5 Search for target driver genes and functional analysis

The target driver genes of the studied miRNAs were searched in the *miRTarBase* public database (http://mirtarbase.mbc.nctu.edu.tw/) (Chou et al., 2016), using just interactions experimentally validated by strong evidence (qRT-PCR, Luciferase reporter assay, Western blot and Microarray). Identification of genes that are regulated by at least two miRNAs in common, among the nine studied and with statistical significance, was made using *MiRTargetLink* tool (Hamberg et al., 2016). Enrichment analysis of differentially expressed miRNAs and shared target genes were conducted in KEGG and Gene Ontology (Ashburner et al., 2000; Gene Ontology Consortium, 2021). Interaction networks and enriched pathways were constructed using cnetplot() function in *R* or *Cytoscape v.3.9.1* (Shannon et al., 2003).

## 3 Results

### 3.1 Samples characteristics

A total of 108 individual samples were recruited for this study. After clinical and histopathological assessments, bacilloscopy and qPCR, all the LP were classified according to Ridley and Jopling’s criteria of disease and reactions, 33 BT-TT patients and 26 BL-LL patients. The 49 non-LP samples were obtained from healthy household contacts and tested negative for all tests for leprosy. There were no significant differences between LP and non-LP in sex (*p* > 0.05, χ^2^ test), but there were in terms of age (*p* ≤ 0.05, Wilcoxon test) (Table 1). BL-LL patients had positive bacilloscopy, while BT-TT patients had negative bacilloscopy, which is in accordance with the expected characteristics of these leprosy subforms. Only three BT-TT samples were non-reactive to detection of antibodies against phenolic glycolipid I (PGL-I) and four BL-LL samples were undetermined for qPCR (Table 1).

**TABLE 1:** Clinical characteristics of samples.

Variable	Non-LP (*n* = 49)	LP (*n* = 59)	*p*-value
Sex			0.42^a^
Male	25	24	
Female	24	34	
Age	37.40 ± 16.11	45.89 ± 16.44	0.01^b^
Bacilloscopy		BT-TT BL-LL	<0.0001^c^
Negative	49	33 0	
Positive	0	0 26	
Anti-PGL-I		BT-TT BL-LL	0.25^c^
Non-reactive	49	3 0	
Positive	0	30 26	
RLEP-qPCR		BT-TT BL-LL	0.37^c^
Undetermined	49	4 1	
Positive	0	29 25	

^a^ χ2 test.

^b^ Wilcoxon test.

^c^ Fisher exact test.

### 3.2 Expression profile of miRNAs in blood samples and evaluation of their potential as biomarker

**FIGURE 1:**
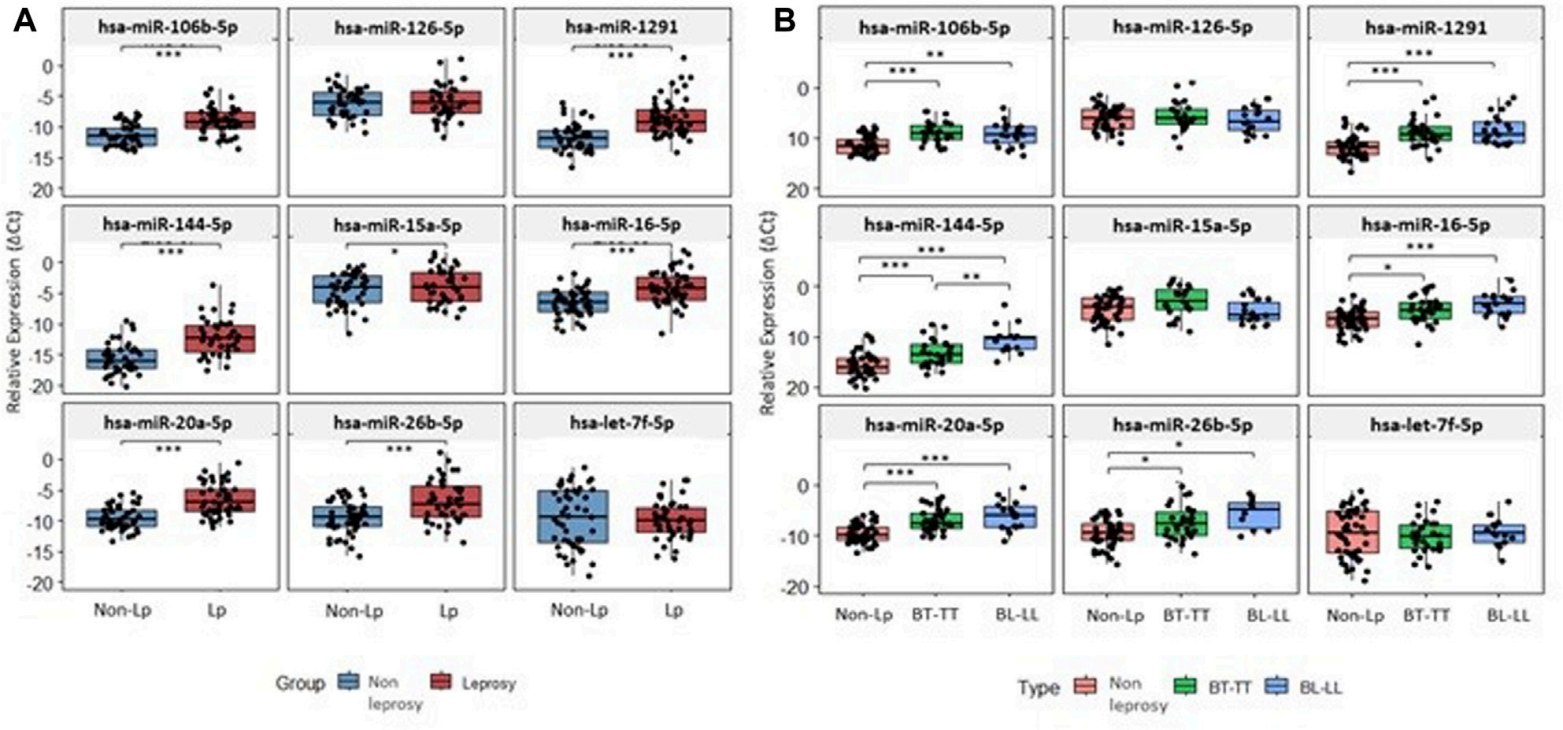
Expression level of studied miRNAs. **(A)** Expression level of miRNAs between non-LP and LP. **(B)** Expression level of miRNAs between non-LP and BT-TT and BL-LL poles. *p-value < 0.05; **p-value < 0.001; ***p-value < 0.0001; *p-value* adjusted by FDR correction.

Of all nine miRNAs studied, seven (*hsa-miR-106b-5p*, *hsa-miR-1291*, *hsa-miR-144-5p*, *hsa-miR-15a-5p*, *hsa-miR-16-5p*, *hsa-miR-20a-5p* and *hsa-miR-26b-5p*) were significantly upregulated in LP (*p* ≤ 0.05) (Figure 1A). *Hsa-miR-106b-5p*, *hsa-miR-1291*, *hsa-miR-144-5p*, *hsa-miR-16-5p*, *hsa-miR-20a-5p* and *hsa-miR-26b-5p* were upregulated in both poles, and *hsa-miR-144-5p* was upregulated in BL-LL form when compared with BT-TT pole (Figure 1B). Similarly, these seven miRNAs were upregulated in male and female LP (*p* ≤ 0.05). In male patients, *hsa-miR-1291* was upregulated in both poles (*p* ≤ 0.05) (Supplementary Figure S1A). Likewise, *hsa-miR-144-5p* and *hsa-miR-20a-5p* were upregulated in BL-LL pole (*p* ≤ 0.05). *Hsa-miR-15a-5p, hsa-126-5p* and *hsa-let7f-5p* were shown to be downregulated in male LP, without statistical significance. In female patients, *hsa-106b-5p*, *hsa-miR-144-5p*, *hsa-miR-16-5p*, *hsa-miR-20a-5p* and *hsa-miR-26b-5p* were all upregulated at both poles (*p* ≤ 0.05). Furthermore, all these miRNAs, except *hsa-miR-106b-5p*, were more upregulated in BL-LL pole when compared to BT-TT pole (*p* ≤ 0.05) (Supplementary Figure S1B). The expression values (2^−ΔCt^) and Fold Change values are presented in Supplementary Tables S2, S3 in the Supplementary Material.

In LP, miRNA expression comparisons were also made for two of the diagnostic methods used: Anti-PGL-I (>0.295 and <0.295) (Gobbo et al., 2022) and RLEP-qPCR (Ct > 35 and <35) (Da Silva et al., 2018; Da Silva et al., 2021), finding that *hsa-miR-20a-5p* was significantly upregulated in patients with PGL-I values > 0.295 (Supplementary Figure S3A). However, when the expression of the miRNAs was compared according to sex, it was found no significant expression in male patients, but in female patients, four miRNAs, *hsa-miR-106b-5p*, *hsa-miR-16-5p*, *hsa-miR-20a-5p* and *hsa-miR-26b-5p*, were significantly upregulated in patients with PGL-I > 0.295 (Supplementary Figure S3B). For RLEP-qPCR, only *hsa-miR-15a-5p* showed significant upregulated in patients with Ct > 35 compared to Ct < 35 (Supplementary Figure S4). No miRNA was differentially expressed when comparisons were made according to sex.

**FIGURE 2:**
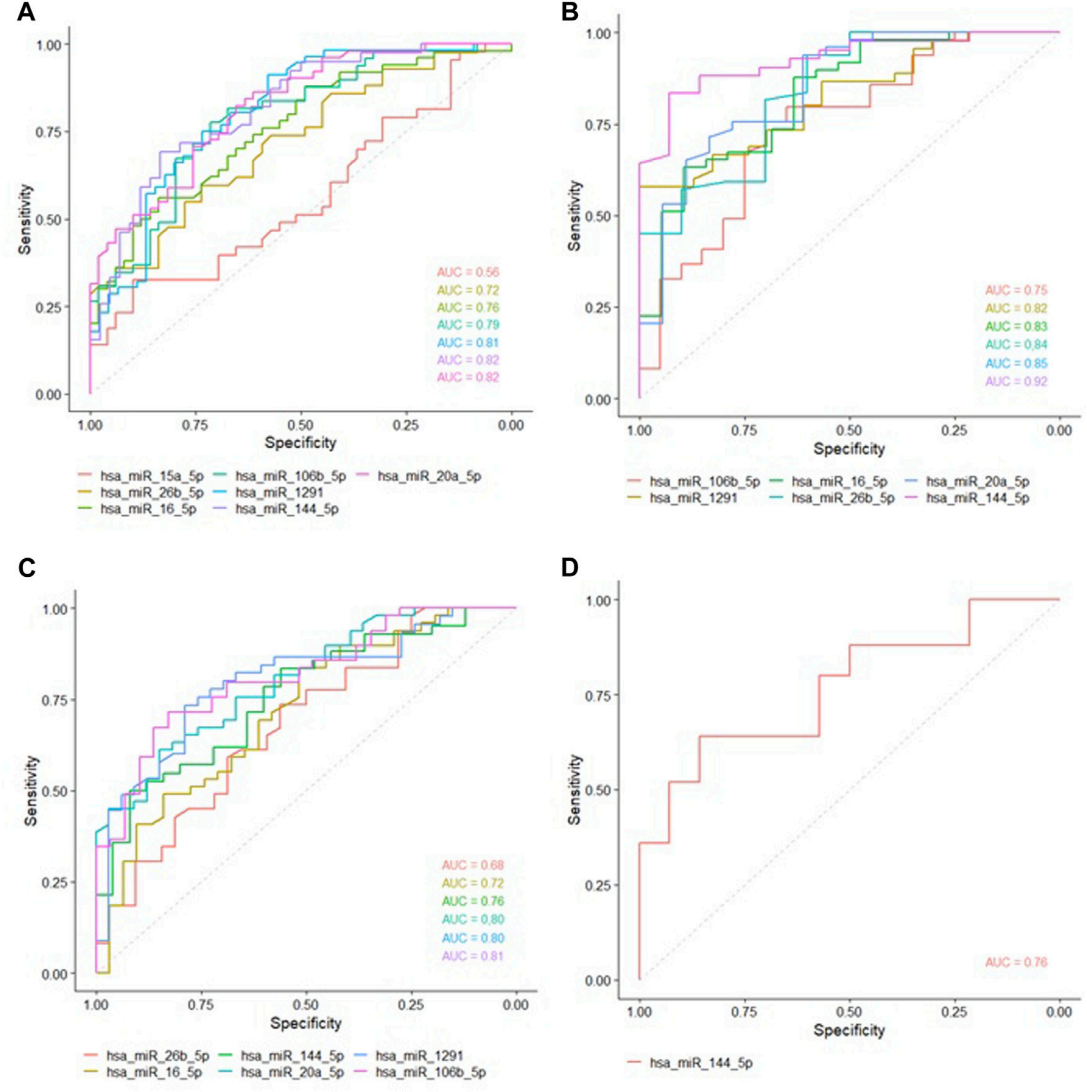
ROC curves of studied miRNAs with statistically significant expression between the groups. Area Under the Curve (AUC) with at least 0.75 was considered as good ability in discriminating between comparations. **(A)** ROC curve between non-LP and LP. **(B)** ROC curve between non-LP and BL-LL poles. **(C)** ROC curve between non-LP and BT-TT poles. **(D)** ROC curve between BT-TT and BL-LL poles.

*Hsa-miR-16-5p, hsa-miR-106b-5p, hsa-miR-1291, hsa-miR-144-5p* and *hsa-miR-20a-5p*, were able to discriminate, with great accuracy, between non-LP and LP (AUC>0.75) (Figure 2A) and between non-LP and BL-LL pole (Figure 2B). *Hsa-miR-106b-5p*, *hsa-miR-1291*, *hsa-miR-144-5p* and *hsa-miR-20a-5p* were able to discriminate, with great accuracy, between non-LP and BT-TT pole (AUC>0.75) (Figure 2C). Comparisons between BT-TT and BL-LL poles, only showed *hsa-miR-144-5p* with AUC>0.75 (Figure 2D). Same comparisons were performed by sex and they are showed in Supplementary Figures S5, S6 and Supplementary Tables S4, S5. MiRNAs that showed a significant differential expression between all comparisons and with an AUC>0.75, were proposed as possible diagnostic biomarkers for leprosy (Table 2; Supplementary Tables S4, S5).

**TABLE 2:** Potential miRNA biomarkers for leprosy.

miRNA	Non-LP vs. LP	*p-value*	AUC	Non-LP vs. BT-TT	*p-value*	AUC	Non-LP vs. BL-LL	*p-value*	AUC	BT-TT vs. BL-LL	*p-value*	AUC
*hsa-miR-144-5p*	✔	2.9e-07	0.82	✔	8.1e-04	0.76	✔	8.1e-04	0.81	✔	7.9e-03	0.76
*hsa-miR-20a-5p*	✔	2.7e-09	0.82	✔	5.7e-06	0.80	✔	5.7e-06	0.81	✖	ns	—
*hsa-miR-1291*	✔	1.2e-07	0.81	✔	2.5e-05	0.80	✔	2.5e-05	0.82	✖	ns	—
*hsa-miR-106b-5p*	✔	1.1e-06	0.79	✔	1.3e-05	0.82	✔	1.3e-05	0.79	✖	ns	—
*hsa-miR-16-5p*	✔	2.8e-06	0.76	✖	2.4e-03	0.72	✔	2.4e-03	0.76	✖	ns	—
*hsa-miR-26b-5p*	✖	1.1e-04	0.72	✖	7e-02	0.68	✖	7e-02	0.72	✖	ns	—
*hsa-miR-15a-5p*	✖	0.03	0.56	✖	ns	0.68	✖	ns	—	✖	ns	—
*hsa-miR-126-5p*	✖	ns	—	✖	ns	—	✖	ns	—	✖	ns	—
*hsa-let7f-5p*	✖	ns	—	✖	ns		✖	ns	—	✖	ns	—

*ns: no significance.

### 3.3 Target gene identification

**FIGURE 3:**
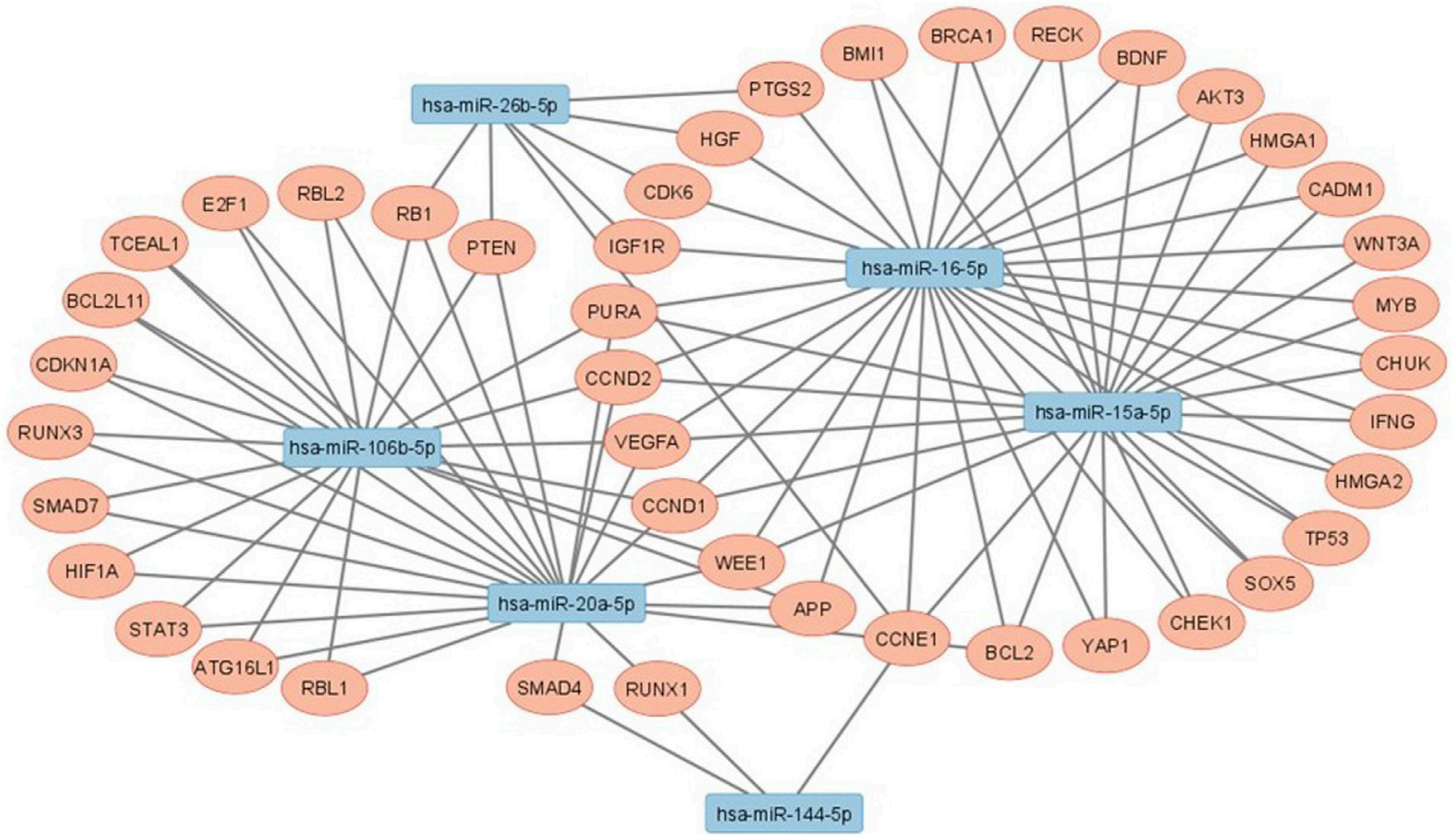
Interaction networks between studied miRNAs that had statistically significant results and their target genes.

Genes that are regulated by at least two miRNAs with significant differential expression between non-LP and LP were investigated using *MiRTargetLink* tool (Hamberg et al., 2016), founding in total 43 regulated genes (Table 3). Enrichment analyzes showed processes that are related mainly to the development to cell cycle and several pathways that are related to leprosy such as apoptosis, autophagy/mitophagy, and immune system like Th1, Th2 and Th17 cell differentiation (Supplementary Figure S5). Interaction networks between miRNAs and target genes was constructed using *Cytoscape v.3.9.1* (Shannon et al., 2003) (Figure 3).

**TABLE 3:** List of genes that were potentially targeted by two or more differentially expressed miRNAs.

Gene	MiRNA	N° miRNAs
*CCND1*	*hsa-miR-15a-5p*, *hsa-miR-16-5p*, *hsa-miR-20a-5p*, *hsa-miR-106b-5p*	4
*CCND2*	*hsa-miR-15a-5p*, *hsa-miR-16-5p*, *hsa-miR-20a-5p*, *hsa-miR-106b-5p*	4
*CCNE1*	*hsa-miR-15a-5p*, *hsa-miR-16-5p*, *hsa-miR-26b-5p*, *hsa-miR-144-5p*	4
*PURA*	*hsa-miR-15a-5p*, *hsa-miR-16-5p*, *hsa-miR-20a-5p*, *hsa-miR-106b-5p*	4
*CCND1*	*hsa-miR-15a-5p*, *hsa-miR-16-5p*, *hsa-miR-20a-5p*, *hsa-miR-106b-5p*	4
*VEGFA*	*hsa-miR-15a-5p*, *hsa-miR-16-5p*, *hsa-miR-20a-5p*, *hsa-miR-106b-5p*	4
*WEE1*	*hsa-miR-15a-5p*, *hsa-miR-16-5p*, *hsa-miR-20a-5p*, *hsa-miR-106b-5p*	4
*APP*	*hsa-miR-16-5p*, *hsa-miR-20a-5p*, *hsa-miR-106b-5p*	3
*BCL2*	*hsa-miR-15a-5p*, *hsa-miR-16-5p*, *hsa-miR-20a-5p*	3
*PTEN*	*hsa-miR-20a-5p*, *hsa-miR-26b-5p*, *hsa-miR-106b-5p*	3
*RB1*	*hsa-miR-20a-5p*, *hsa-miR-26b-5p*, *hsa-miR-106b-5p*	3
*AKT3*	*hsa-miR-15a-5p*, *hsa-miR-16-5p*	2
*ATG16L1*	*hsa-miR-20a-5p*, *hsa-miR-106b-5p*	2
*BCL2L11*	*hsa-miR-20a5p*, *hsa-miR-106b-5p*	2
*BDNF*	*hsa-miR-15a-5p*, *hsa-miR-16-5p*	2
*BMI1*	*hsa-miR-15a-5p*, *hsa-miR-16-5p*	2
*BRCA1*	*hsa-miR-15a-5p*, *hsa-miR-16-5p*	2
*CADM1*	*hsa-miR-15a-5p*, *hsa-miR-16-5p*	2
*CDK6*	*hsa-miR-16-5p*, *hsa-miR-26b-5p*	2
*CDKN1A*	*hsa-miR-20a-5p*, *hsa-miR-106b-5p*	2
*CHEK1*	*hsa-miR-15a-5p*, *hsa-miR-16-5p*	2
*CHUK*	*hsa-miR-15a-5p*, *hsa-miR-16-5p*	2
*E2F1*	*hsa-miR-20a-5p*, *hsa-miR-106b-5p*	2
*HGF*	*hsa-miR-16-5p*, *hsa-miR-26b-5p*	2
*HIF1A*	*hsa-miR-20a-5p*, *hsa-miR-106b-5p*	2
*HMGA2*	*hsa-miR-15a-5p*, *hsa-miR-16-5p*	2
*IFNG*	*hsa-miR-15a-5p*, *hsa-miR-16-5p*	2
*IGF1R*	*hsa-miR-16-5p*, *hsa-miR-26b-5p*	2
*MYB*	*hsa-miR-15a-5p*, *hsa-miR-16-5p*	2
*PTGS2*	*hsa-miR-16-5p*, *hsa-miR-26b-5p*	2
*RBL1*	*hsa-miR-20a-5p*, *hsa-miR-106b-5p*	2
*RBL2*	*hsa-miR-20a-5p*, *hsa-miR-106b-5p*	2
*RECK*	*hsa-miR-15a-5p*, *hsa-miR-16-5p*	2
*RUNX1*	*hsa-miR-20a-5p*, *hsa-miR-144-5p*	2
*RUNX3*	*hsa-miR-20a-5p*, *hsa-miR-106b-5p*	2
*SMAD4*	*hsa-miR-20a-5p*, *hsa-miR-144-5p*	2
*SMAD7*	*hsa-miR-20a-5p*, *hsa-miR-106b-5p*	2
*SOX5*	*hsa-miR-15a-5p*, *hsa-miR-16-5p*	2
*STAT3*	*hsa-miR-20a-5p*, *hsa-miR-106b-5p*	2
*TCEAL1*	*hsa-miR-20a-5p*, *hsa-miR-106b-5p*	2
*TP53*	*hsa-miR-15a-5p*, *hsa-miR-16-5p*	2
*WNT3A*	*hsa-miR-15a-5p*, *hsa-miR-16-5p*	2
*YAP1*	*hsa-miR-15a-5p*, *hsa-miR-16-5p*	2

## 4 Discussion

Compelling evidence indicates that circulating miRNAs might serve as crucial biomarkers across various conditions, including several cancer types (Wang et al., 2016; O’Bryan et al., 2017; Chen et al., 2019; Filipów and Ł, 2019; Hasanzadeh et al., 2019), cardiovascular disease (Corsten et al., 2010; Cheng et al., 2014; Wang et al., 2017a), infectious diseases (Furci et al., 2013; Kaur et al., 2018; Poore et al., 2018; Silva et al., 2021), and neurology, particularly in diagnosing and predicting Alzheimer’s disease (Wiedrick et al., 2019). This potential positions miRNAs as a promising diagnostic and prognostic tool for numerous illnesses. Although there are current constraints, exploring miRNAs as biomarkers in diverse conditions continues to be a remarkable avenue of research. Leprosy manifests slowly, and its symptoms are challenging to detect in the initial stages of infection.

Generally, leprosy diagnosis is delayed and there are few research groups or health professionals skilled of making an early diagnosis. Diagnosis delay can lead to the development of the most advanced stages of the disease, thus increasing the probabilities of transmission of the infectious agent and committing the quality of life of those affected by the disease. For this reason, the study of miRNAs, and pathways that they are involved can generate new knowledge to understand the complex genetic and immune regulation of leprosy, thus directing new approaches to prevention, diagnosis and treatment, through the use of miRNAs as possible biomarkers of the disease. Our study found that *hsa-miR-144-5p*, *hsa-miR-20a-5p*, *hsa-miR-1291* and *hsa-miR-106b-5p* are good candidates for leprosy early biomarkers. In addition, several evaluated miRNAs are capable of accurately distinguishing between various of the comparisons characterized in this study (Table 2; Supplementary Tables S4, S5). Besides, *hsa-miR-20a-5p* was upregulated in LP with Anti-PGL-I > 0.295, and *hsa-miR-106b-5p*, *hsa-miR-16-5p*, *hsa-miR-20a-5p* and *hsa-miR-26b-5p* were significantly upregulated in female LP with PGL-I > 0.295. The PGL-I fraction is part of the cell envelope of *M. leprae* and induces the production of the humoral specific response against PGL-I detected in serum of patients (Foss et al., 1993). When the antibody is present at high levels the infection can be supposed to be active, particularly during reactional episodes, which constitute a very common complication in the evolution of leprosy (Goulart et al., 2002). Therefore, these miRNAs could also act as biomarkers for the active form and for reactive episodes of the disease. For RLEP-qPCR, *hsa-miR-15a-5p* showed significant upregulated in LP with Ct > 35 compared to Ct < 35. Lower Cts values generally belong to multibacillary cases, while higher Cts values to paucibacillary cases (Azevedo et al., 2017; Gobbo et al., 2022). *Hsa-miR-15a-5p* showed lower expression (although without statistical significance) in BL-LL patients, when compared to BT-TT patients, so its potential use as biomarker to discriminate between the poles of the disease may be considered.

Household contacts of leprosy patients constitute a population of risk to be monitored for the early detection of leprosy (Cardona-Castro et al., 2005). Several studies in Brazil have detected cases of leprosy in asymptomatic household contacts. One of them detected a high frequency of *M. Leprae* in 23.89% of asymptomatic home contacts evaluated by RLEP-qPCR (Gama et al., 2018). Another study reported an increase of 16 times in the new case detection rate after training a specialist to carry out active search actions with neurodermatological analysis among household contacts in low endemicity areas (Bernardes et al., 2017). It was also found that prevalence in schoolchildren, in endemic areas of Amazonian populations, was 17 times greater than the officially reported rate (Pedrosa et al., 2018). Official data from Brazil show that about 50% of the population living in 19 of the 27 states are exposed to either high or hyperendemic rates of infection. The estimated number of hidden cases of leprosy is likely to be up to eight times higher than the prevalence in the area at any given time (Salgado et al., 2018b). Additionally, there have been limited studies evaluating the genetic and epigenetic profile of household contacts. Consequently, it is imperative to conduct further evaluation to identify new early diagnostic biomarkers as well as potential therapeutic targets.

### 4.1 Sex bias in leprosy

In general, a male preponderance over female has been reported in various epidemiological studies on leprosy, although in recent years the difference between sex has decreased (Peters and Eshiet, 2002; Van Veen et al., 2006; Padhi and Pradhan, 2015; Cáceres-Durán, 2023). Several factors traditionally attributed to women, especially in countries like India, such as low status, more limited mobility, analphabetism, and poor knowledge of the disease, could be responsible for the underreporting of cases of women affected by leprosy (Sarkar and Pradhan, 2016). The genetic background between men and women may also be involved in the immune response against *M. leprae*. To date, no large-scale study has reported the contribution of genetic factors to sex bias in leprosy. Although social and behavioral factors could contribute to male bias in leprosy, the role of variants in sex chromosomes has not been studied (Fava et al., 2020). It is known that the X chromosome carries several genes related to the immune system that could contribute to the sexual bias observed in leprosy (Jaillon et al., 2019). A study in Brazil found that polymorphisms in the *TLR1* gene were associated with greater protection against leprosy in females (Niitsuma et al., 2018). Another study, also in Brazil, associated polymorphisms in *TNFα* gene as protector against LL pole in women. Increased production of TNFα results from decrease in IL-10, a proinflammatory cytokine that inhibits synthesis of anti-inflammatory cytokines and activation of T cells (Santos et al., 2002). Because of this sex bias it would be important to find sex-specific diagnostic biomarkers for leprosy.

Our study showed, for the first time, a differential expression of miRNAs by sex in LP. The expression of *hsa-miR-1291* was significantly more elevated in male LP (Supplementary Table S4) than in females LP (Supplementary Table S5), suggesting it as a potential specific biomarker for males. Additionally, the expression of *hsa-miR-16-5p* and *hsa-miR20a-5p* was significantly more elevated in female LP (Supplementary Table S5), suggesting them as a potential specific biomarker for females. Also, although there was no statistical significance, *hsa-miR-126-5p*, *has-15a-5p and has-let7f-5p* were downregulated in male LP. This result coincides with those previously published by (Salgado et al., 2018a), where the differentially expressed miRNAs were downregulated. It should be noted that all samples sequenced in the miRnoma were from male LP. In general, *hsa-miR-144-5p* appear to have the best potential as biomarkers to discriminate between the comparisons in all LP (Table 2).

The expression of miRNAs in leprosy patients was found to be differentially regulated between males and females which may contribute to the sexual bias observed in this disease. This sexual bias could also be influenced by the hormonal dependence of *M. leprae*, or by hormonal imbalances during the disease (Rée et al., 1981). In fact, individuals with the LL form of leprosy often present alterations in various endocrine and sexual hormones, which could be related to the disease’s progression (Dabi et al., 2023). Therefore, the crosstalk between sex hormones and immune effectors emerges as one of the main candidate drivers of gender differences in infectious disease susceptibility, as previously reported (Guerra-Silveira and Abad-Franch, 2013).

### 4.2 Apoptosis, autophagy, mitophagy and cell cycle are dysregulated in LP

**FIGURE 4:**
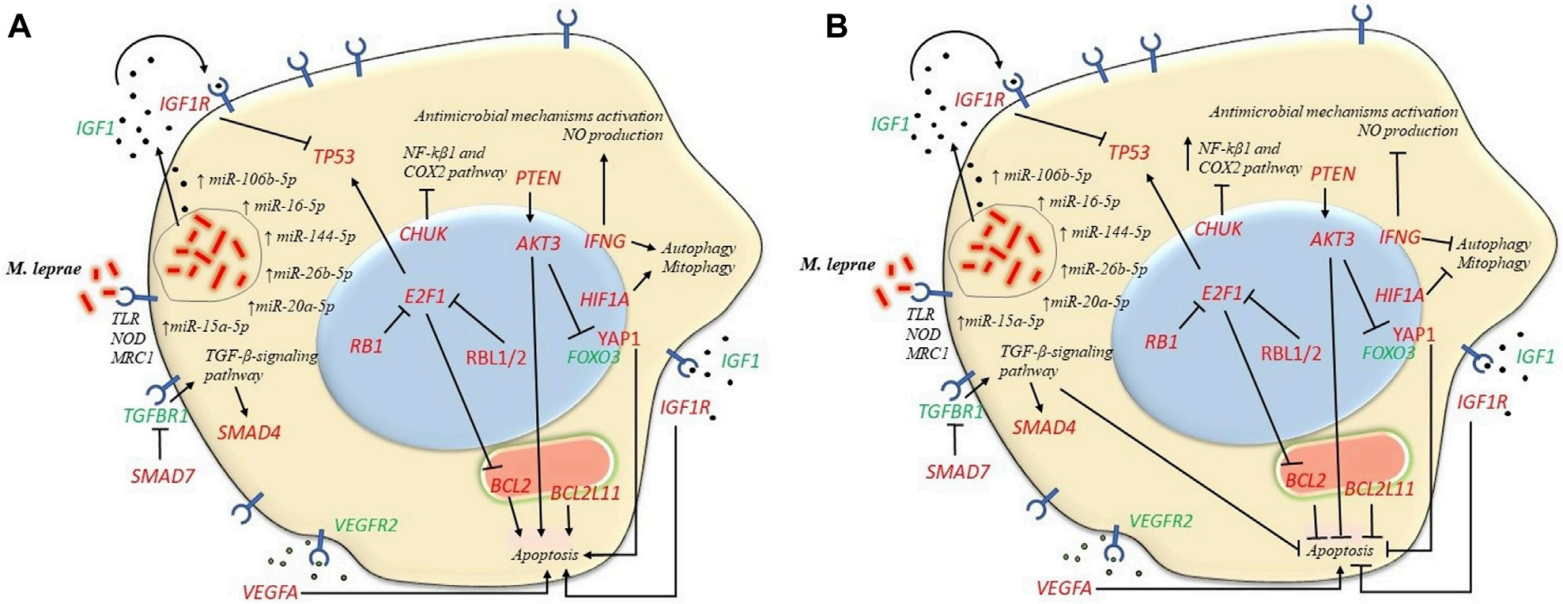
Expression of microRNAs strictly regulates various cellular pathways such as apoptosis, autophagy and mitophagy. In the first place the engulfment of *M. leprae* by phagocytosis could be triggered by *IGF1R* which is targeted by several miRNAs. This process can result in the suppression of macrophages' ability to eliminate the mycobacteria, ultimately impairing their microbicidal function. **(A)** Pro-apoptotic profile in BT-TT form. MiRNAs that target anti-apoptotic genes *BCL2*, *AKT3* and *PTEN* were more upregulated in BT-TT form consequently leading to a pro-apoptotic profile. Furthermore, miRNAs that target pro-apoptotic genes *BCL2L11, YAP1* and *SMAD4/SMAD7* and autophagy/mitophagy-related genes like *IFNG* and *HIF1A* were less upregulated in this form, leading to a pro-apoptotic profile and a more autophagic/mitophagic profile in the BT-TT pole. **(B)** Anti-apoptotic profile on BL-LL form. Conversely, the differences in miRNAs expression between disease forms lead to an anti-apoptotic profile and the inhibition of autophagy/mitophagy in BL-LL form. Genes in red are targeted by the upregulated miRNAs in our study and genes in green are not direct targets of the studied miRNAs.

Functional enrichment analysis revealed participation of pathways involved in apoptosis, autophagy and mitophagy. Although the role of apoptosis during *M. Leprae* infection is not yet clear, this is a defense mechanism against pathogens. There are studies that demonstrate pro-apoptotic effects (Hernandez et al., 2003; Ajith et al., 2005; Quaresma et al., 2010; Quaresma et al., 2014) and others that demonstrate anti-apoptotic effects (Hasan et al., 2006; Rodrigues et al., 2010). In addition, there also are studies that suggest a modulation of apoptosis mechanisms in the spectrum of the disease with low levels of apoptosis in the LL pole and high levels in the TT pole (Brito de Souza et al., 2010). Comparisons of lesions between polar forms revealed that apoptosis is more frequent in TT patients suggesting that activation of apoptosis could act containing the multiplication of mycobacteria (Walsh et al., 2004). High levels of apoptosis could favor the control of the bacillary load observed in TT patients and low apoptosis rate in LL patients could contribute to the persistence of mycobacteria (Ajith et al., 2005). MiRNAs that regulate anti-apoptotic genes such as *BCL2* and *AKT3* were found to be upregulated in LP (Figure 4), which could be associated with a pro-apoptotic state. In particular, when comparing the expression of miRNAs between the two poles, *hsa-miR-15a-5p* showed a 4-fold lower expression in BL-LL form, suggesting a lower downregulation of its target genes *BCL2* and *AKT3* in these patients. This might result in an anti-apoptotic profile in this pole which is associated with a decrease in immune response. Conversely, it was found that *hsa-miR-16-5p* expression, that also regulates *BCL2* and *AKT3*, was increased by 2.58-fold in the BL-LL pole, which may contribute to a pro-apoptotic profile in these patients. Also, *AKT* inhibits the expression of pro-apoptotic genes *YAP1* and *FOXO3*, while stimulating the expression of the anti-apoptotic gene *MDM4* (Salgado et al., 2018a). *YAP1* is also targeted by *hsa-miR-15a-5p* and *hsa-miR-16-5p.* This deregulation leads to a lack of control in the apoptosis related pathways in LP (Figures 4A, B).

In addition to this, *BCL2L11* gene, which encodes a member of the BCL2 family that promotes apoptosis (O’Connor et al., 1998), is regulated by *hsa-miR-20a-5p*. This miRNA was found to be expressed 12-fold higher in the BL-LL form leading to an anti-apoptotic state in this form, which is associated with a less effective immune response. Bim protein is a crucial factor in initiating apoptosis and shaping immune responses (Sionov et al., 2015). Additionally, by inhibiting *SMAD4* and *SMAD7*, *hsa-miR-20a-5p, hsa-miR-106b-5p* and *hsa-miR-144-5p* could inhibit apoptosis by activating the TGF-β signaling pathway (Xiao et al., 2012; Yao et al., 2018). Notably, *hsa-miR-144-5p* was found to be expressed 38-fold higher in BL-LL form. Besides, *VEGFR2* is recognized for its involvement in *Mycobacterium tuberculosis* (*Mtb*) dissemination by inducing angiogenesis, whereas *VEGFA*, its corresponding ligand (Salgado et al., 2018a) is targeted by *hsa-miR-15a-5p*, *hsa-miR-16-5p*, *hsa-miR-20a-5p* and *hsa-miR-106b-5p*, playing an important role in the regulation of apoptosis. Upon *M. leprae* infection, *IFGR1*, the receptor of *IGF1*, is activated in macrophages and Schwann cells, which in turn inhibits apoptosis and promotes cell survival. This leads to the production and secretion of IGF, which further stimulates cell survival and pathogen proliferation (Batista-Silva et al., 2016). In general, all these findings indicate a dysregulation at various levels in the apoptosis process in leprosy patients (Figures 4A, B).

Autophagy has been associated with immune responses against intracellular pathogens such as *Mtb* and it can be induced by IFN-γ to inhibit intracellular survival of mycobacteria (Harris et al., 2009). *Hsa-miR-15a-5p* and *hsa-miR-16-5p* regulate *IFNG* gene affecting the process of autophagy. Conversely, Th2 cytokines IL-4 and IL-13 inhibit autophagy in macrophages (Harris et al., 2009). The role of autophagy in the immunopathogenesis of leprosy is not fully understood but *M. leprae* infection can alter host cell autophagy as an immune escape mechanism. Proinflammatory cytokines can induce autophagy in TT pole lesions, while BCL2 family members can alter autophagy induction in LL pole lesions (Silva et al., 2017). Studies have shown that T1R patients had hypo-regulation of autophagy associated with hyper-regulation of *TLR2* and *MLST8* (de Mattos Barbosa et al., 2018).

Mitophagy is important for the removal of redundant and damaged mitochondria to maintain cell survival and viability in response to injury, trauma, and infection. Ineffective removal of damaged mitochondria can cause chronic systemic inflammation and the development of inflammatory diseases (Gkikas et al., 2018). *HIF1A*, a transcription factor that responds to low oxygen availability and plays a regulatory role in response to a variety of molecular signals of infection and inflammation and induce autophagy (Gladek et al., 2017; Santos and Andrade, 2017), is targeted by *hsa-miR-20a-5p* and *hsa-miR-106b-5p* in LP. As previously mentioned, *hsa-miR-20a-5p* was found to be increased 12-fold in the BL-LL pole, thereby altering mitophagy in these patients. Mitochondrias are known to play a role in the immune response and have been implicated in various infectious diseases, including mycobacterial infections (de Souza and Cavalcante, 2022). Furthermore, mitophagy also plays a role in the differentiation of M1 or M2 macrophages, which could provide a link between the immune response and the metabolic phenotype observed in leprosy. Additionally, mitochondrias play a role in the nerve damage detected in leprosy patients (van Hooij and Geluk, 2021).

Cell cycle regulation is a crucial process for maintaining proper cellular function. *AKT3* plays a vital role in this process, as well as in apoptosis (Chang et al., 2014; Chang et al., 2015; Wang et al., 2017b). It was showed that that overexpression of *hsa-miR-16-5p* and knockdown of *AKT3* led to a significantly decreased number of cells in the S phase and a significantly increased number of cells in the G0/G1 phase, coinciding with the suppression of cell survival (Wang et al., 2020). Additionally, disruption of *AKT3* has been shown to significantly reduce neuron viability and axon length and can actively participate in neuronal death and pathologies associated with uncontrolled cell growth (Diez et al., 2012). Impairment of nerve function in leprosy often results in chronic deformities and disabilities. Cumulative nerve damage affects sensory, motor, and autonomic systems, and is typically characterized by nerve enlargement. LL patients with clinical nerve function impairment are at a high risk of further nerve function deterioration (Wilder-Smith and Van Brakel, 2008).

Other genes related to cell cycle are targeted by several miRNAs studied. The tumor suppressor gene *PTEN* is targeting by *hsa-miR-20a-5p*, *hsa-miR-26b-5p* and *hsa-miR-106b-5p*. This gene is involved in critical cellular processes such as survival, proliferation, energy metabolism and cellular architecture (Huang et al., 2012; Song et al., 2012). *Hsa-miR-15a-5p* and *hsa-miR-16-5p*, have been found to target the *TP53* gene, which is not only crucial for the immune response but it also plays a role in antibacterial activity. As well, it was showed that inhibition of *IGF1R* reduces *TP53* and *MDM2* translation through a gene-specific mechanism (Xiong et al., 2007). *IGF1R* is targeting by *hsa-miR-16-5p, hsa-miR-26b-5p* which also leads to downregulation of the *TP53* gene in LP affecting apoptosis (Figure 4). These miRNAs were increased by 10 and 15-fold, respectively, in the BL-LL pole. A study on *Mtb* found that TP53-deficient macrophages failed to control the mycobacteria, manifesting in a lower rate of apoptosis and greater intracellular survival of the pathogen (Lim et al., 2020). In addition, the PI3K/AKT/PTEN signaling pathway plays a role in regulation of apoptosis and autophagy (Paul-Samojedny et al., 2015).

Cyclins (*CCND1, CCND2* and *CCNE1*), cyclin-dependent kinases (*CDKN1A* and *CDK6*) and *RB1* are also being target of several miRNAs studied. All of them contribute to activation of downstream *E2F* transcription factors, which in turn can cause uncontrolled cell proliferation and ectopic cell divisions (Johnson et al., 2016). *E2F* targets are involved in cell cycle regulation, glycolysis, fatty acid metabolism and mitochondrial functions (Bracken et al., 2004; Benevolenskaya and Frolov, 2015).

Overall, this evidence suggests that leprosy disrupts the cell cycle at various stages and may also modulate apoptosis, autophagy, and mitophagy, potentially contributing to pathogen survival.

### 4.3 Immune effector cells activation

For a long time, the main interpretation of the host defense response was based on the characterization of the Th1/Th2 paradigm. However, new approaches have emerged that have changed the interpretation of this paradigm in the polar forms of the disease, especially with the identification of other subtypes of T lymphocytes such as Th9, Th17, Th22 and Tregs (de Sousa et al., 2017). Th1-Th2 and Th17 differentiation pathways were shown to be enriched in our study with several genes being regulated by miRNAs evaluated. *IFNG* is targeted by *hsa-miR16-5p*, that presented a 10-fold higher expression in BL-LL patients than in BT-TT patients. A greater production of IFN-γ has been demonstrated in BT-TT individuals during primary response to infection by *M. leprae* (de Almeida-Neto et al., 2015). Several studies have reported that IFN-γ and TNF-α have an important role in immune protection. IFN-γ activates antimicrobial mechanisms, inducing inducible NO synthase production, leading to production of nitric oxide, an important microbicide that destroys the *bacillus* by releasing free radicals (Moubasher et al., 1998). IFN-γ also induces macrophages to produce TNF-α, leading to activation of these same cells. It has been shown that TNF-α is present in serum of BT-TT patients and absent in serum of BL-LL patients, indicating that *bacillus* destruction and granuloma formation must be related to presence of this cytokine (Murray et al., 1985; Madan et al., 2011).

LL form is associated with a greater number of lesions with presence of foamy macrophages and globes. There is a predominance of a Th2 lymphocyte response in this polar form which induces the production of cytokines such as IL-4, IL-10 and TGF-β that inactivate the microbicidal response of macrophages facilitating the *bacillus* survival. This type of response produced by Th2 cells negatively regulates the Th1 response by inhibiting the microbicidal response of macrophages at this pole (de Sousa et al., 2017).

Participation of Th17 cells and their main product IL-17 in immunology of leprosy was confirmed. Some studies reported the presence of Th17 in ENL reactions expressing low IL-17 in the skin (Martiniuk et al., 2012). Others studies have reported that the IL-17F isomer is associated with reversal reactions of leprosy (Chaitanya et al., 2012). It was also reported that Th17 cells with the IL-17A signature and its IL-17C, D, F, E, and RORC isoforms are more associated with BT-TT form both in skin lesions and in PBMC cultures induced by *M. leprae*, suggesting its differential role in patients (Saini et al., 2013). Also, a positive correlation between IFN-γ and IL-17 was established, as well as the differentiation of the frequency of these cells between TT and LL individuals (de Almeida-Neto et al., 2015).

Upregulated miRNAs *hsa-miR-20a-5p* and *hsa-miR-106b-5p* control *SMAD7* gene and *hsa-miR-15a-5p* and *has-16-5p* control *CHUK* gene. Low levels of *SMAD7* may result in an increase in *TGFBR1* gene, with more TGF-β capture contributing to the immunosuppressive profile in LP. *CHUK* inhibits the NF-kβ1 and COX2 inflammatory pathway (Salgado et al., 2018a) (Figure 4).

Of the nine miRNAs evaluated, *hsa-let7f-5p* was the only one that was shown to be downregulated (although with not statistical significance) in all LP. Studies in *Mtb*-infected macrophages also revealed this RNA being downregulated. *Has-let-7f-5p* targets A20, a feedback inhibitor of the NF-kβ pathway. It was shown that *Mtb*-infected mice have a lower expression of *hsa-let7f* and a higher expression of A20 during infection progression. Macrophages with decreased expression of A20 showed reduced survival of *Mtb* and concomitantly produced lower levels of TNF, IL-1B, and nitrites (Kumar et al., 2015). These observations suggest that a comparable mechanism may also be involved in the pathogenesis of leprosy.

## 5 Final considerations

A good diagnostic test will recognize *M. leprae* infected people at risk of developing diseases or leading to their spread. Hence, identification of new blood-based biomarker in leprosy is a need to also switch from leprosy management to prevention of infection. The advantages of blood biomarkers lie in their high accessibility and practicality as blood is an easily obtainable and non-invasive sample compared to a skin or nerve biopsy. This makes blood biomarkers a valuable tool for leprosy diagnosis especially in the early stages for patients who are asymptomatic.

In conclusion, it is known that altered expression of miRNAs occurs in various types of diseases, cancer being one of the most studied. Research has provided a better understanding of pathophysiological mechanisms of different diseases at the molecular level, some of which have shown peculiar patterns in miRNA expression that allow their molecular classification. In addition, differential expression of these molecules in diseases have been object of study for search new biomarkers with prognostic, diagnostic and therapeutic potential. However, studies with miRNAs in infectious diseases are emerging, they are still not enough, mainly in leprosy. We found that *hsa-miR-144-5p*, *hsa-miR-20a-5p*, *hsa-miR-1291* and *hsa-miR-106b-5p* are potential candidates for leprosy early biomarkers. These miRNAs seem to play an important role in the pathogenesis of the disease and could be used as new biomarkers for early diagnosis in order to decrease or stop its transmission and prevent the development of the most advanced stages of the disease.

Limitations: This research constitutes an initial investigation into the modulation of miRNA within the framework of the host response. Several limitations warrant careful consideration. Firstly, the participants' comorbidities were not taken into account, as many were unaware of the potential existence of other underlying conditions. Additionally, specific forms of leprosy were excluded from our study due to the limited availability of samples. Moreover, a larger sample size is essential to establish a more robust correlation between the assessed miRNAs and disease progression.

## Data Availability

The original contributions presented in the study are included in the article/Supplementary Material, further inquiries can be directed to the corresponding authors.
