# Supplementary material for: MicroRNA biomarkers in leprosy: insights from the Northern Brazilian Amazon population and their implications in disease immune-physiopathology
Source: Front Genet. 2024 Jan 25;15:1320161. doi: 10.3389/fgene.2024.1320161 (PMC10854011; doi:10.3389/fgene.2024.1320161)
Supplement: Supplementary file 1 [file Table1.DOCX]

Supplementary Material

**MicroRNA Biomarkers in Leprosy: Insights from the Northern Brazilian Amazon Population and their Implications in Disease Immune-Physiopathology**

Miguel Ángel Cáceres-Durán^1*^; Pablo Pinto^1-2^; Leandro Magalhães^1^; Tatiane Piedade de Souza^1^; Angelica Gobbo^3^; Josafá Gonçalves Barreto^3^; Moises Batista da Silva^2-4^; Patrícia Fagundes da Costa^2^; Claudio Salgado^2^; Ândrea Ribeiro-dos-Santos^1*^

^1^Laboratório de Genética Humana e Médica, Instituto de Ciências Biológicas (ICB), Universidade Federal do Pará (UFPA), Belém, Brazil

^2^ Laboratório de Dermato-Imunologia, ICB, UFPA, Marituba, Brazil

^3^ Laboratório de Epidemiologia Espacial, UFPA, Castanhal, Brazil

^4^ Laboratório de Patologia Geral, UFPA, Belém, Brazil

*** Correspondence:**
[macdur@gmail.com](mailto:macdur@gmail.com), [akelyufpa@gmail.com](mailto:akelyufpa@gmail.com)

**Table S1. Primers used in the study**

| **Primer** | **Sequence** |
| --- | --- |
| *hsa-miR-106b-5p-F* | 5’-TAAAGTGCTGACAGTGCAGAT-3’ |
| *hsa-miR-126-5p-F* | 5’-CATTATTACTTTTGGTACGCG-3’ |
| *hsa-miR-1291-F* | 5’-TGGCCCTGACTGAAGACCAGCAGT-3’ |
| *hsa-miR-144-5p-F* | 5’-GGATATCATCATATACTGTAAG-3’ |
| *hsa-miR-15a-5p-F* | 5’-TAGCAGCACATAATGGTTTGTG-3’ |
| *hsa-miR-16-5p-F* | 5'-TAGCAGCACGTAAATATTGGCG-3’ |
| *hsa-miR-20a-5p-F* | 5'-TAAAGTGCTTATAGTGCAGGTAG-3’ |
| *hsa-miR-26b-5p-F* | 5'-TTCAAGTAATTCAGGATAGGT-3’ |
| *hsa-let7f-5p-F* | 5'-TGAGGTAGTAGATTGTATAGTT-3’ |
| *Reverse qPCR* | 5’-CGAGGAAGAAGACGGAAGAAT-3’ |
| *RNU6B-F* | 5’- CGCAAGGATGACACGCAAATT CGTGAAGCGTTCCATATTTTT-3’ |
| *RNU24-F* | 5’-ATTTGCTATCTGAGAGATGGTGATGACATTTTAAACCAC CAAGATCGCTGATGCA-3’ |
| *Universal stem-loop* | 5’-GAAAGAAGGCGAGGAGCAGATCGAGGAAGAAGACGGAAGAATGTGCGTCTCGCCTTCTTTCNNNNNNNN-3’ |

**Supplementary table 2.** ΔCt of miRNAs

| Sample | Case_  Non-LP | Type | Endogenous | miR_15a_5p | miR_126_5p | miR_1291 | miR_let7f_5p | miR_144_5p | miR_106b_5p | miR_16_5p | miR_20a_5p | miR_26b_5p |
| --- | --- | --- | --- | --- | --- | --- | --- | --- | --- | --- | --- | --- |
| C1 | Non-LP | Non-LP | 14,10 | 6,45 | 4,61 | 10,61 | 13,10 | 14,69 | 11,53 | 4,75 | 9,17 | 8,81 |
| C2 | Non-LP | Non-LP | 13,08 | 5,74 | 8,22 | 12,75 | 15,53 | 17,24 | 13,07 | 7,93 | 13,41 | 9,55 |
| C3 | Non-LP | Non-LP | 13,82 | 6,61 | 9,58 |  | 13,03 | 18,41 | 13,92 | 6,03 | 8,37 | 6,71 |
| C4 | Non-LP | Non-LP | 14,08 | 6,74 | 9,13 |  | 10,28 | 18,29 | 13,43 | 4,02 | 7,11 | 8,06 |
| C5 | Non-LP | Non-LP | 12,94 | 2,17 | 3,38 |  | 9,80 |  | 11,95 | 4,89 | 8,34 | 5,06 |
| C6 | Non-LP | Non-LP | 15,55 | 8,46 | 8,92 | 11,37 | 17,19 |  | 13,44 | 9,52 | 10,72 | 13,73 |
| C7 | Non-LP | Non-LP | 15,00 | 4,00 |  | 13,45 | 12,83 | 19,69 | 13,61 | 5,92 | 9,84 | 10,42 |
| C8 | Non-LP | Non-LP | 14,74 | 5,11 |  | 13,95 | 17,12 | 20,15 | 13,68 | 9,97 | 11,61 | 13,57 |
| C9 | Non-LP | Non-LP | 14,54 | 0,67 | 6,81 | 12,83 | 18,93 |  | 11,84 | 10,58 | 11,56 | 14,50 |
| C10 | Non-LP | Non-LP | 12,16 | 1,87 |  |  | 13,06 |  | 10,33 | 7,66 | 9,52 | 5,23 |
| C11 | Non-LP | Non-LP | 14,43 | 4,15 | 10,93 | 10,94 | 15,89 | 18,91 | 13,11 | 7,13 | 10,96 | 11,11 |
| C12 | Non-LP | Non-LP | 14,88 | 8,05 | 8,48 | 10,28 | 14,80 | 15,35 | 11,11 | 6,48 | 10,64 | 10,23 |
| C13 | Non-LP | Non-LP | 13,29 | 1,54 | 7,49 | 10,81 | 6,27 |  | 11,89 | 6,11 | 10,02 | 5,73 |
| C14 | Non-LP | Non-LP | 14,13 | 3,23 | 9,61 | 10,38 | 11,56 | 15,58 | 11,46 | 4,77 | 9,46 | 8,47 |
| C15 | Non-LP | Non-LP | 13,77 | 3,70 | 5,45 | 11,54 | 14,05 | 17,37 | 11,58 | 6,34 | 10,63 | 10,94 |
| C16 | Non-LP | Non-LP | 14,89 | 3,57 | 6,19 | 11,18 | 14,94 | 18,50 | 11,83 | 7,42 | 11,31 | 11,16 |
| C18 | Non-LP | Non-LP | 14,02 | 6,13 |  | 13,14 | 13,25 | 17,14 | 13,09 | 6,18 | 10,39 | 9,57 |
| C19 | Non-LP | Non-LP | 15,84 | 6,12 | 7,58 | 12,15 | 14,43 | 16,44 | 12,22 | 7,75 | 11,11 | 10,31 |
| C20 | Non-LP | Non-LP | 15,77 | 7,14 | 7,54 | 11,84 | 9,41 | 14,48 | 11,24 | 3,46 | 7,99 | 4,98 |
| C21 | Non-LP | Non-LP | 14,30 | 9,36 | 9,41 | 13,72 | 13,51 | 16,25 | 13,08 | 6,99 | 8,68 | 9,27 |
| C22 | Non-LP | Non-LP | 14,03 | 2,30 | 7,21 | 13,41 | 14,33 | 17,11 | 12,87 | 8,39 | 10,86 | 10,68 |
| C23 | Non-LP | Non-LP | 14,20 | 2,36 | 9,81 | 14,62 | 14,05 | 17,23 | 13,85 | 8,56 | 11,80 | 11,47 |
| C24 | Non-LP | Non-LP | 12,77 | 2,16 |  | 14,42 | 10,70 |  | 12,63 | 11,17 | 12,49 | 8,60 |
| C25 | Non-LP | Non-LP | 14,22 | 2,92 |  | 14,13 | 16,62 | 17,77 | 13,08 | 10,71 | 12,59 | 12,52 |
| C26 | Non-LP | Non-LP | 15,75 | 8,13 | 5,96 | 11,73 | 13,43 | 15,17 | 11,04 | 7,34 | 10,34 | 8,56 |
| C27 | Non-LP | Non-LP | 16,48 | 1,83 | 5,51 | 11,19 | 11,61 | 14,88 | 10,60 | 5,19 | 9,35 | 7,60 |
| C28 | Non-LP | Non-LP | 14,72 | 11,51 | 3,85 | 11,55 | 9,12 | 16,01 | 12,34 | 8,56 | 11,23 | 10,16 |
| C29 | Non-LP | Non-LP | 14,42 | 2,08 | 5,10 | 12,70 | 8,60 | 14,32 | 11,99 | 9,29 | 10,87 | 6,25 |
| C30 | Non-LP | Non-LP | 14,43 | 3,05 | 8,37 | 12,44 | 5,55 | 17,08 | 11,87 | 7,02 | 10,32 | 10,14 |
| C31 | Non-LP | Non-LP | 16,54 | 1,96 | 6,09 | 10,08 | 6,55 | 16,28 | 11,47 | 7,44 | 10,68 | 10,53 |
| C32 | Non-LP | Non-LP | 17,43 | 9,34 | 5,56 | 10,86 | 3,25 | 13,18 | 10,14 | 4,64 | 8,36 | 6,91 |
| C33 | Non-LP | Non-LP | 15,14 | 0,43 | 3,54 | 13,31 | 5,06 | 16,23 | 13,05 | 6,54 | 10,43 | 13,14 |
| C34 | Non-LP | Non-LP | 15,12 | 1,73 | 6,45 | 13,00 | 4,24 | 14,92 | 13,50 | 6,71 | 10,16 | 10,92 |
| C35 | Non-LP | Non-LP | 14,26 | 1,52 | 5,03 | 14,25 | 7,84 | 17,62 | 12,81 | 7,94 | 11,62 | 13,42 |
| C36 | Non-LP | Non-LP | 17,90 | 6,81 | 4,06 | 8,27 | 4,86 | 14,27 | 7,68 | 5,65 | 8,82 | 9,41 |
| C37 | Non-LP | Non-LP | 17,49 | 3,47 | 2,26 | 7,84 | 4,65 | 14,22 | 8,99 | 5,81 | 6,97 | 10,85 |
| C38 | Non-LP | Non-LP | 17,50 | 0,96 | 2,95 | 7,74 | 3,40 | 16,89 | 9,89 | 5,20 | 7,76 | 9,04 |
| C39 | Non-LP | Non-LP | 18,45 | 7,83 | 3,90 | 6,05 | 5,93 | 15,11 | 8,64 | 7,84 | 7,67 | 11,91 |
| C40 | Non-LP | Non-LP | 16,30 | 6,59 |  | 9,74 | 8,26 | 18,41 | 11,53 | 10,47 | 10,30 | 15,69 |
| C41 | Non-LP | Non-LP | 19,83 | 4,23 | 1,56 | 7,07 | 4,23 | 13,32 | 8,14 | 4,35 | 6,68 | 8,95 |
| C42 | Non-LP | Non-LP | 16,35 | 5,16 | 3,79 | 9,55 | 3,91 | 12,19 | 9,24 | 3,69 | 7,19 | 6,82 |
| C43 | Non-LP | Non-LP | 16,58 | 4,40 | 3,36 | 7,49 | 3,62 | 10,83 | 8,55 | 2,87 | 6,29 | 6,03 |
| C44 | Non-LP | Non-LP | 16,88 | 1,73 | 4,74 | 12,28 | 7,90 | 17,00 | 11,13 | 8,42 | 9,66 | 12,91 |
| C45 | Non-LP | Non-LP | 17,39 | 6,65 | 4,44 | 12,19 | 5,10 | 14,03 | 10,15 | 5,24 | 7,86 | 9,49 |
| C46 | Non-LP | Non-LP | 18,17 | 4,16 | 7,54 | 16,60 | 1,25 | 12,23 | 7,96 | 1,81 | 5,91 | 5,84 |
| C47 | Non-LP | Non-LP | 21,93 | 3,71 |  | 11,16 | 3,48 | 10,12 | 8,47 | 2,63 | 5,51 | 6,05 |
| C48 | Non-LP | Non-LP | 18,20 | 4,05 |  | 13,11 | 5,54 | 13,73 | 9,86 | 4,91 | 7,58 | 9,06 |
| C49 | Non-LP | Non-LP | 16,49 | 7,04 | 7,54 | 13,83 | 6,35 |  | 8,95 | 6,30 | 9,07 | 7,68 |
| C50 | Non-LP | Non-LP | 16,75 | 5,77 | 8,22 | 13,49 | 2,64 | 9,52 | 8,60 | 4,72 | 8,49 | 7,99 |
| C17 | LP | BT-TT | 15,78 |  | 8,49 | 12,27 | 10,98 | 15,83 | 9,78 | 3,99 | 8,60 | 7,70 |
| P1 | LP | BT-TT | 13,27 | 3,99 |  | 2,90 | 8,61 |  |  |  | 8,43 | 4,63 |
| P10 | LP | BT-TT | 20,86 |  | 11,76 | 8,32 | 7,67 | 13,69 | 8,36 | 1,66 | 5,91 | 4,98 |
| P11 | LP | BT-TT | 15,78 | 4,56 |  | 9,40 | 12,98 | 16,08 | 12,29 | 6,32 | 10,21 | 10,14 |
| P13 | LP | BT-TT | 18,18 | 4,98 | 6,76 | 9,29 | 7,21 | 11,46 | 7,15 | 0,09 | 3,34 | 3,86 |
| P15 | LP | BT-TT | 14,70 | 2,90 | 5,94 | 7,33 | 12,47 | 14,97 | 11,34 | 7,11 | 10,18 | 9,94 |
| P16 | LP | BT-TT | 17,45 | -0,91 | 6,87 | 11,37 | 12,65 | 15,32 | 10,26 | 6,49 | 9,54 | 11,81 |
| P18 | LP | BT-TT | 17,04 | -0,30 | 5,01 | 10,65 | 9,44 | 14,15 | 8,81 | 5,19 | 7,69 | 7,57 |
| P21 | LP | BT-TT | 16,88 | -0,26 | 3,19 | 8,78 | 14,32 | 15,40 | 9,63 | 6,43 | 7,25 | 10,69 |
| P22 | LP | BT-TT | 15,09 |  | 7,16 | 6,27 | 13,01 | 15,24 | 9,62 | 7,02 | 8,11 | 7,89 |
| P24 | LP | BT-TT | 15,22 | 1,18 | 4,08 | 8,50 | 16,20 | 17,04 | 10,76 | 11,54 | 10,14 | 13,56 |
| P25 | LP | BT-TT | 16,80 | 3,18 |  | 10,43 | 9,58 | 10,74 | 10,01 | 3,88 | 5,85 | 6,39 |
| P26 | LP | BT-TT | 16,30 |  | 6,10 | 9,17 | 8,41 | 11,25 | 8,98 | 3,88 | 5,53 | 7,49 |
| P27 | LP | BT-TT | 17,63 | 6,43 | 4,44 | 9,58 | 9,50 | 11,97 | 8,94 | 4,47 | 6,38 | 8,87 |
| P28 | LP | BT-TT | 17,15 | 7,56 | 5,38 | 10,03 | 11,97 | 12,78 | 9,83 | 7,74 | 8,23 | 10,83 |
| P29 | LP | BT-TT | 15,24 |  | 4,29 | 5,25 | 3,31 |  |  | 4,70 | 4,97 | 1,70 |
| P30 | LP | BT-TT | 15,70 | 1,57 |  | 14,14 | 11,44 | 14,43 | 6,87 | 6,22 | 7,68 | 8,82 |
| P3 | LP | BT-TT | 13,10 | 4,36 |  | 1,86 |  |  |  |  | 9,10 |  |
| P4 | LP | BT-TT | 13,73 | 8,97 | 7,76 | 7,63 | 7,57 |  | 7,92 | 6,51 | 10,34 | 9,81 |
| P40 | LP | BT-TT | 18,65 | 0,26 | 2,69 | 8,50 | 10,69 | 13,23 | 8,71 | 4,91 | 6,99 | 8,56 |
| P41 | LP | BT-TT | 19,86 | -0,22 | -0,47 | 8,35 | 10,04 | 8,89 | 4,72 | 1,14 | 2,86 | 1,78 |
| P43 | LP | BT-TT | 18,55 |  |  | 8,61 |  |  | 5,22 | 3,11 | 3,69 | -1,20 |
| P44 | LP | BT-TT | 20,62 | 1,66 | -1,04 | 7,36 | 7,62 | 7,98 | 4,62 | -0,08 | 2,35 | 0,22 |
| P46 | LP | BT-TT | 19,38 | 4,10 | 7,48 | 9,32 | 5,18 | 7,61 | 6,78 | 0,57 | 3,60 | 3,06 |
| P48 | LP | BT-TT | 17,89 | 3,70 | 6,80 | 11,73 | 10,49 | 10,36 | 7,28 | 3,22 | 4,82 | 6,65 |
| P6 | LP | BT-TT | 15,34 |  | 4,66 | 10,22 | 6,13 |  | 7,55 | 4,69 | 8,04 | 6,38 |
| P7 | LP | BT-TT | 17,74 | -1,58 |  | 10,31 | 3,95 | 11,66 | 8,57 | 1,91 | 5,85 | 6,45 |
| P8 | LP | BT-TT | 16,45 |  | 9,71 | 11,33 | 9,46 |  | 10,21 | 3,76 | 8,76 | 9,52 |
| P19 | LP | BT-TT | 13,57 |  | 8,19 | 11,93 | 12,91 | 17,53 | 11,81 | 6,09 | 8,51 | 7,12 |
| P35 | LP | BT-TT | 16,08 | 1,59 | 6,78 | 11,72 | 12,29 | 13,61 | 9,69 | 5,94 | 7,64 | 8,95 |
| P38 | LP | BT-TT | 16,73 | 7,55 | 2,65 | 5,43 | 11,44 | 15,09 | 10,27 | 5,22 | 7,03 | 10,51 |
| P39 | LP | BT-TT | 16,37 | 7,38 |  | 9,83 | 15,79 | 12,75 | 12,00 | 8,49 | 9,03 | 11,85 |
| P5 | LP | BT-TT | 15,52 | 0,88 | 6,12 | -1,21 |  |  |  | 2,85 | 2,93 | 5,35 |
| P2 | LP | BL-LL | 13,10 | 5,26 |  | 1,94 |  |  |  |  |  |  |
| P9 | LP | BL-LL | 18,61 |  | 10,45 | 7,03 | 9,94 | 14,88 | 9,33 | 6,43 | 8,68 | 10,07 |
| P12 | LP | BL-LL | 16,81 | 7,50 | 7,88 | 10,75 | 11,71 | 13,40 | 10,60 | 5,27 | 8,36 | 9,02 |
| P31 | LP | BL-LL | 16,69 | 1,64 | 6,15 | 11,43 | 11,28 | 11,98 | 11,62 | 5,39 | 8,19 | 9,04 |
| P32 | LP | BL-LL | 16,30 | 2,57 | 4,93 | 11,19 | 8,76 | 12,22 | 7,61 | 2,44 | 5,06 | 3,66 |
| P36 | LP | BL-LL | 18,95 | 5,61 | 2,12 | 8,33 | 8,12 | 9,75 | 7,83 | 1,79 | 3,47 | 4,28 |
| P37 | LP | BL-LL | 20,63 | 5,75 | 3,21 | 7,17 | 10,04 | 12,31 | 8,23 | 3,47 | 6,27 | 6,71 |
| P47 | LP | BL-LL | 18,52 | 4,79 | 7,67 |  | 9,41 | 9,95 | 7,56 | 2,45 | 2,76 | 5,61 |
| P49 | LP | BL-LL | 16,78 | 1,47 | 3,78 | 4,24 | 3,33 |  | 7,18 | 2,04 | 3,92 | 2,52 |
| P50 | LP | BL-LL | 22,18 | 2,37 | 4,11 | 6,66 | 5,76 | 6,85 | 3,90 | -1,30 | 0,59 | 1,70 |
| P53 | LP | BL-LL | 15,86 |  |  | 9,48 | 7,33 |  |  | 4,92 |  | 3,30 |
| P54 | LP | BL-LL | 15,46 |  |  | 10,25 |  |  |  |  |  |  |
| P55 | LP | BL-LL | 22,61 | 0,73 | 2,09 |  | 5,74 | 3,68 | 6,06 | -1,95 | 1,85 |  |
| P56 | LP | BL-LL | 19,91 | 3,63 | 5,11 | 9,35 | 10,47 | 7,38 | 8,88 | 0,67 | 4,87 |  |
| P57 | LP | BL-LL | 19,05 |  |  | 5,85 | 9,50 | 9,87 | 8,92 | 2,25 | 4,92 |  |
| P58 | LP | BL-LL | 17,31 | 6,33 | 7,84 | 11,45 | 11,67 | 10,03 | 12,45 | 3,70 | 8,05 |  |
| P59 | LP | BL-LL | 16,52 | 8,02 | 9,54 | 11,08 |  |  | 9,84 |  |  |  |
| P60 | LP | BL-LL | 18,14 |  |  | 10,68 | 12,13 | 10,78 | 11,94 | 4,05 | 8,32 |  |
| P61 | LP | BL-LL | 17,18 | 6,41 | 9,11 | 11,02 |  |  | 9,35 |  |  |  |
| P62 | LP | BL-LL | 15,72 | 6,69 | 5,38 | 11,50 | 15,04 | 12,72 | 13,57 | 7,98 | 11,03 |  |
| P63 | LP | BL-LL | 18,10 | 5,96 | 7,11 | 3,17 | 7,94 |  | 10,09 | 1,74 | 6,32 |  |
| P64 | LP | BL-LL | 15,88 | 7,46 | 9,51 | 4,14 |  |  |  |  |  |  |
| P65 | LP | BL-LL | 18,35 | 5,74 | 6,86 | 8,49 |  |  | 9,62 | 5,75 | 5,75 |  |
| P66 | LP | BL-LL | 16,43 | 7,68 | 8,90 | 8,13 |  |  | 12,03 | 5,88 | 9,57 |  |
| N1 | LP | BL-LL | 17,00 |  |  | 10,93 |  |  |  |  |  |  |


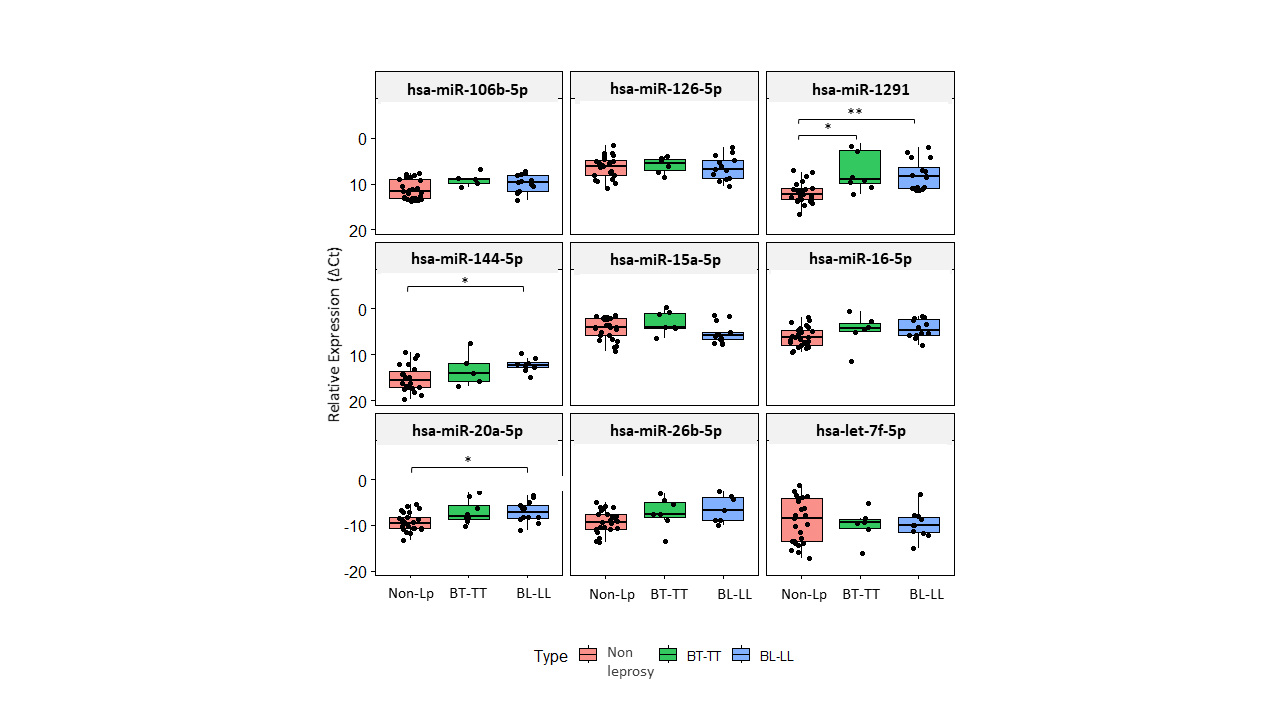

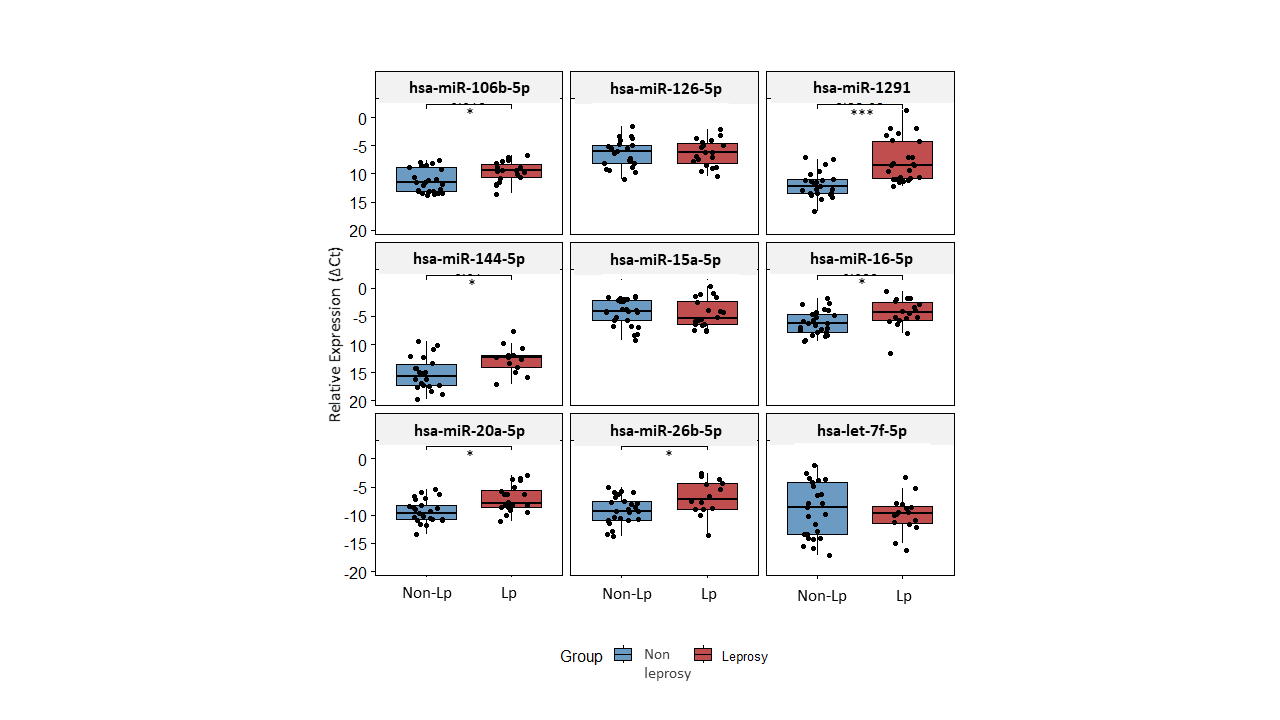
A B

**Supplementary Figure 1**. Expression levels of studied miRNAs in male samples. A) Expression levels of miRNAs among non-LP and LP. B) Expression levels of miRNAs among non-LP and BT-TT and BL-LL poles. *p-value < 0.05; **p-value < 0.001; ***p-value < 0.0001; p-value adjusted by FDR correction.

**
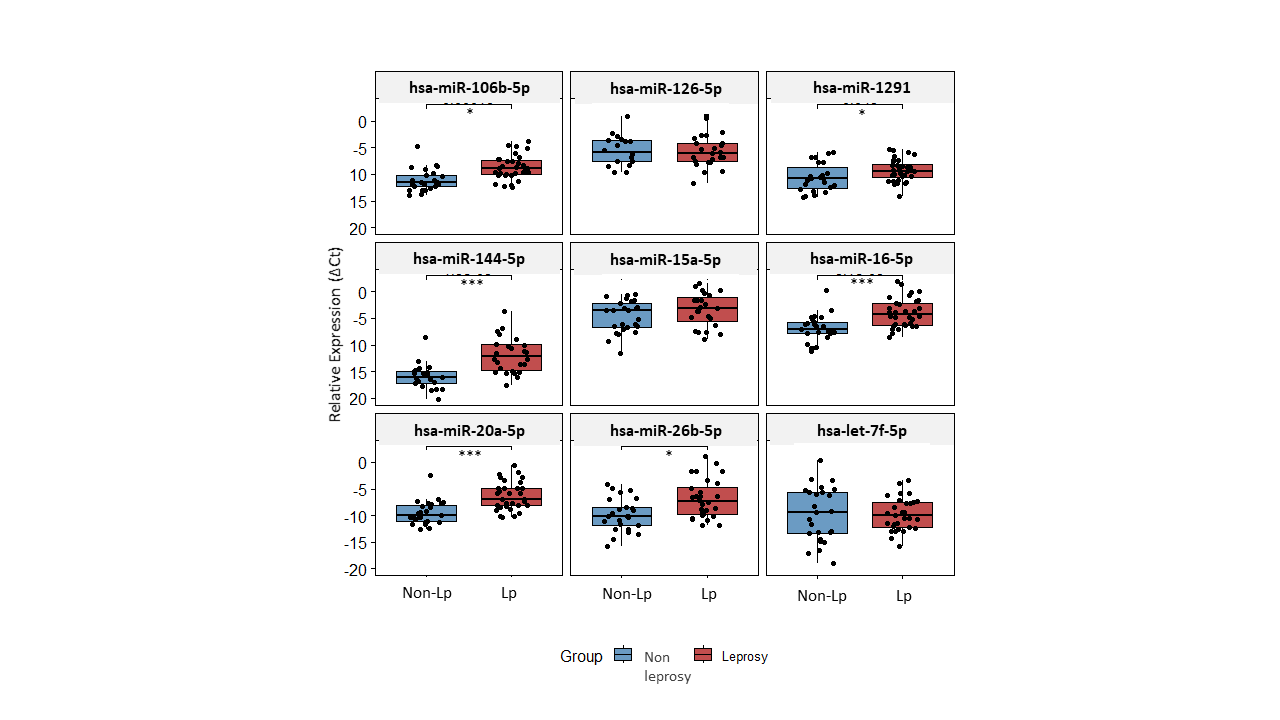

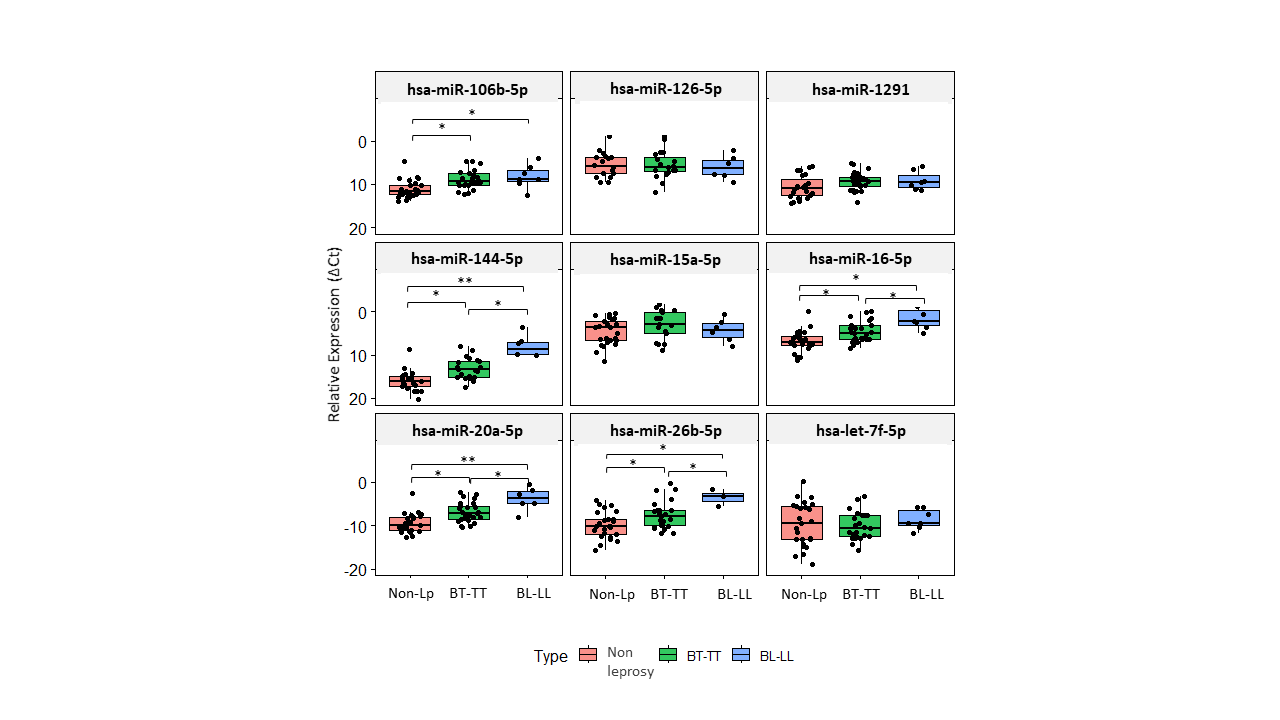
**A B

**Supplementary Figure 2**. Expression levels of studied miRNAs in female samples. A) Expression levels of miRNAs among non-LP and LP. B) Expression levels of miRNAs among non-LP and BT-TT and BL-LL poles. *p-value < 0.05; **p-value < 0.001; ***p-value < 0.0001; p-value adjusted by FDR correction.


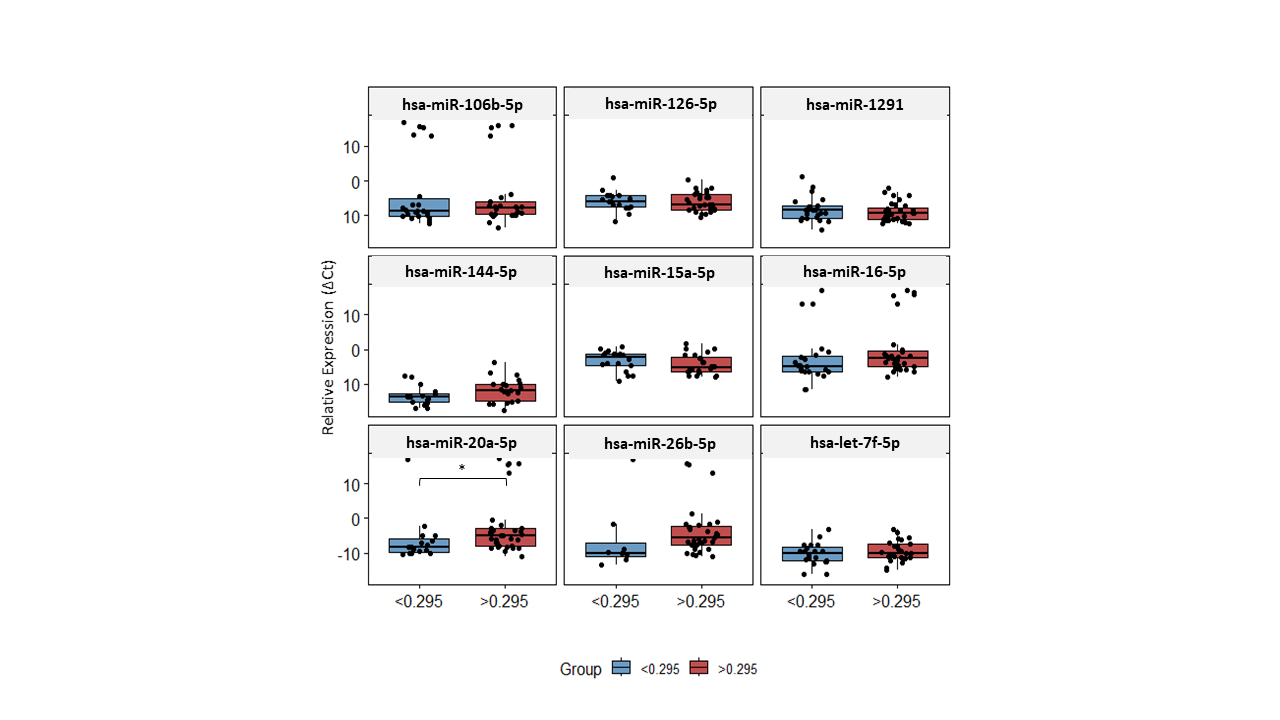

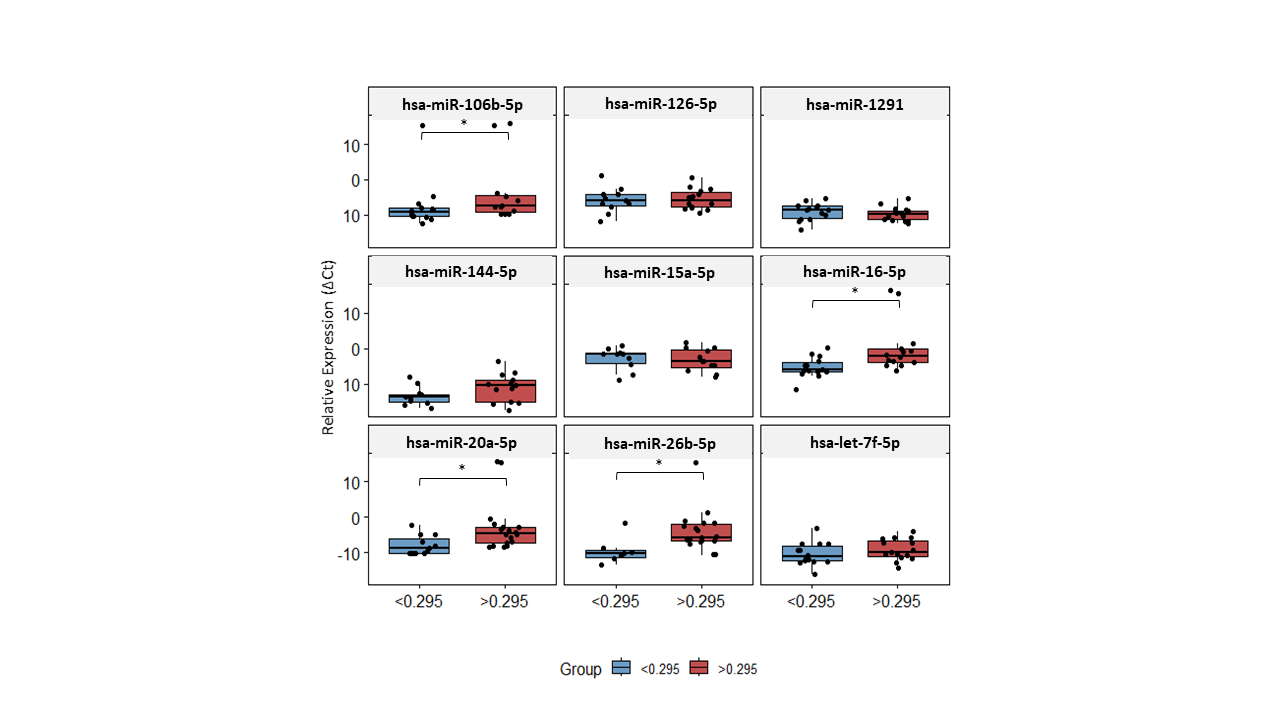
A B

**Supplementary Figure 3**. Expression levels of studied miRNAs in positive PGL-I patients’ samples. A) Expression levels of miRNAs among non-LP and LP. B) Expression levels of miRNAs among non-LP and LP in female samples. *p-value < 0.05; p-value adjusted by FDR correction.


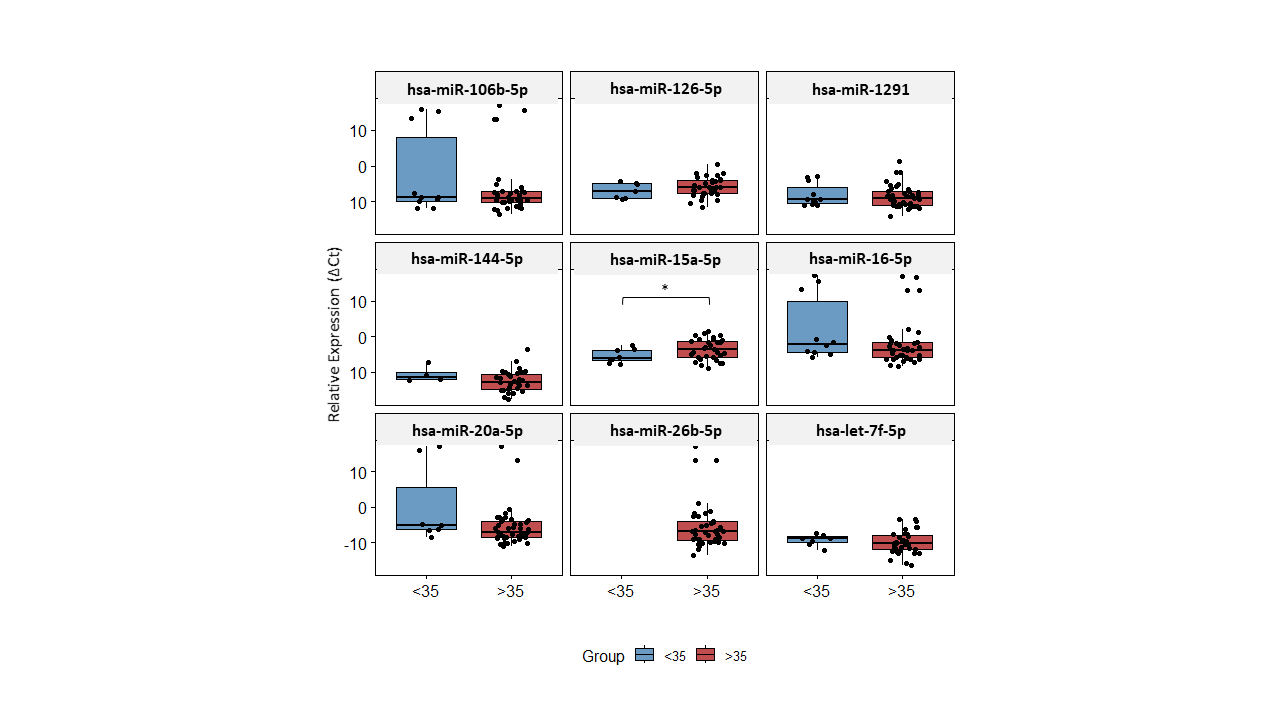


**Supplementary Figure 4**. Expression levels of studied miRNAs in positive RLEP-qPCR patients’ samples. *p-value < 0.05; p-value adjusted by FDR correction.

A

B
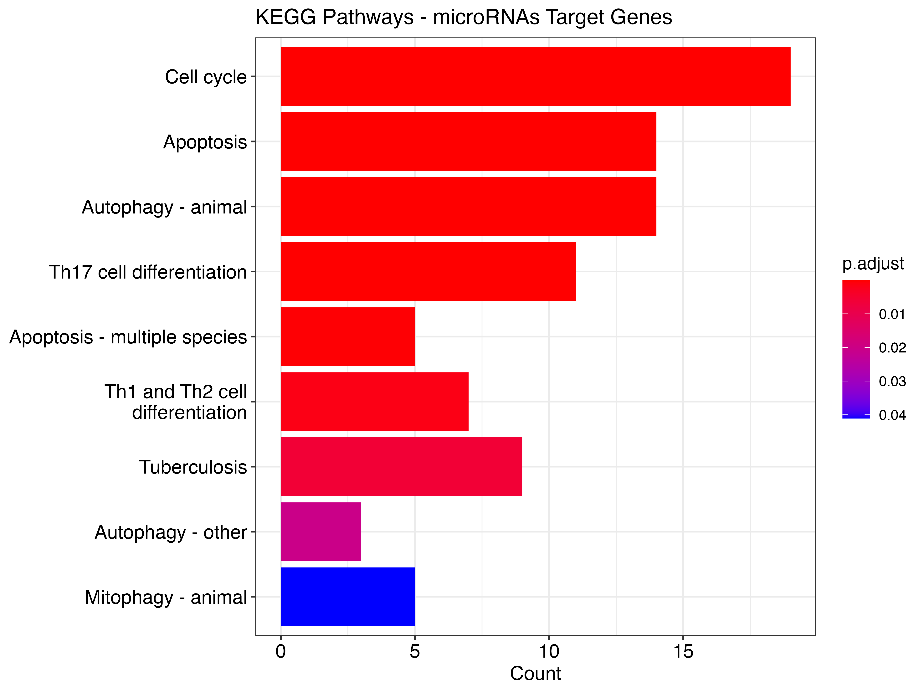

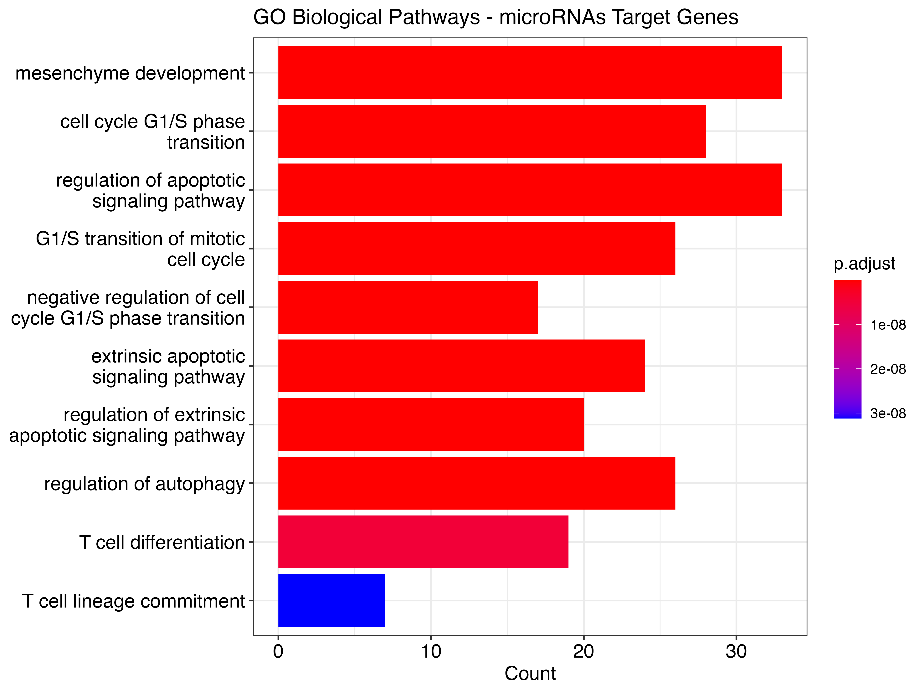


**Supplementary Figure 5**. Functional enrichment analysis of miRNA-mRNA interactions. (A) KEGG enrichment analysis. (B) GO enrichment analysis.

**Supplementary Table 3**. Profile of differential expression for comparisons conduct

|  | LP vs non-LP | | BT-TT vs non-LP | | BL-LL vs non-LP | | BT-TT vs BL-LL | |
| --- | --- | --- | --- | --- | --- | --- | --- | --- |
| miRNAs | Fold Change^*1^ | *padj*^*2^ | Fold Change | *padj* | Fold Change | *padj* | Fold Change | *padj* |
| *hsa-let-7f-5p* | -1.15 |  | -1.41 |  | 1.24 |  | 1.75 |  |
| *hsa-miR-126-5p* | 1.22 | 0.61 | 1.54 | 1 | -1.11 | 1 | -1.7 | 1 |
| *hsa-miR-144-5p* | 11.27 | 2.9 x10-07 | 5.69 | 8.1 x10-04 | 38.22 | 2.3 x10-05 | 6.71 | 7.9x10-03 |
| *hsa-miR-15a-5p* | 1.57 | 0.033 | 2.87 | 0.08 | -1.36 | 0.48 | -3.9 | 0.06 |
| *hsa-miR-20a-5p* | 7.60 | 2.7 x10-09 | 6.01 | 5.7x10-06 | 11.68 | 1.1x10-04 | 1.94 | 0.23 |
| *hsa-miR-26b-5p* | 6.31 | 1.1 x10-04 | 4.81 | 7x10-03 | 15.14 | 6.8x10-03 | 3.15 | 0.17 |
| *hsa-miR-106b-5p* | 4.87 | 1.1x10-06 | 5.50 | 1.3x10-05 | 4.07 | 2.9x10-03 | -1.35 | 0.58 |
| *hsa-miR-1291* | 8.5 | 1.2x10-07 | 7.90 | 2.5x10-05 | 9.33 | 3.3x10-05 | 1.18 | 0.8 |
| *hsa-miR-16-5p* | 5.42 | 2.8x10-06 | 3.79 | 2.4x10-03 | 9.74 | 1.1x10-04 | 2.58 | 0.08 |

*1. Log2 *fold change*; *2 *p* adjusted by FDR


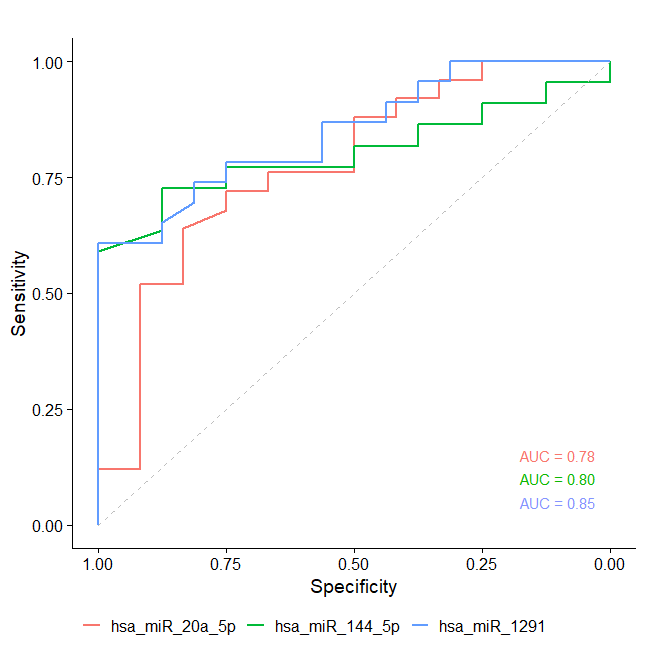
A B


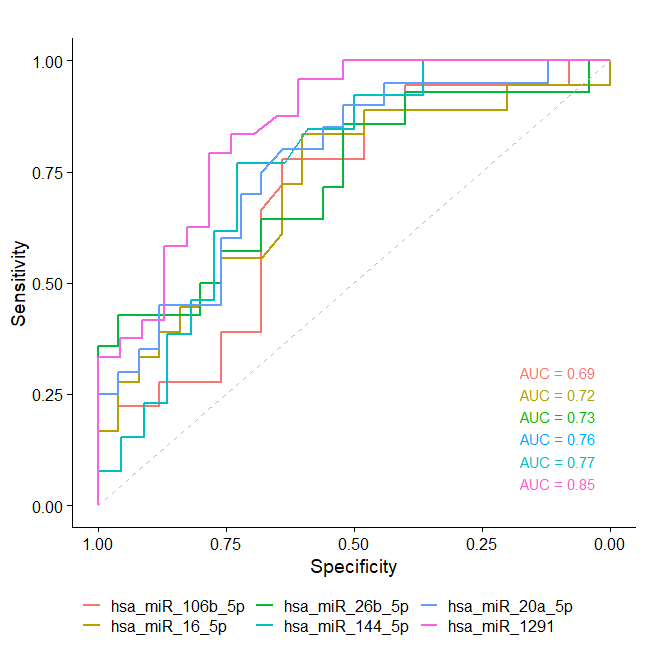


C


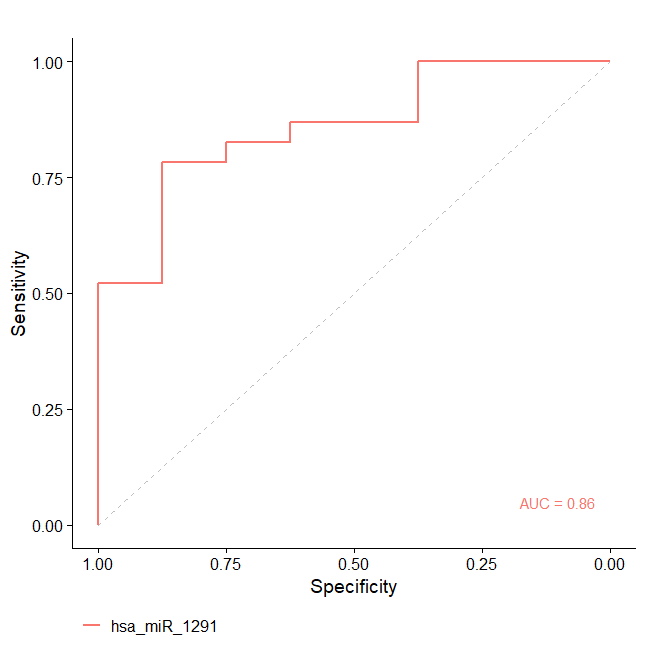


**Supplementary Figure 6.** ROC curves of studied miRNAs with statistically significant expression between the groups in male samples. Area Under the Curve (AUC) with at least 0.75 was considered as good ability in discriminating between comparations. A) ROC curve between non-LP and LP. B) ROC curve between non-LP and BL-LL pole. C) ROC curve between non-LP and BT-TT pole.


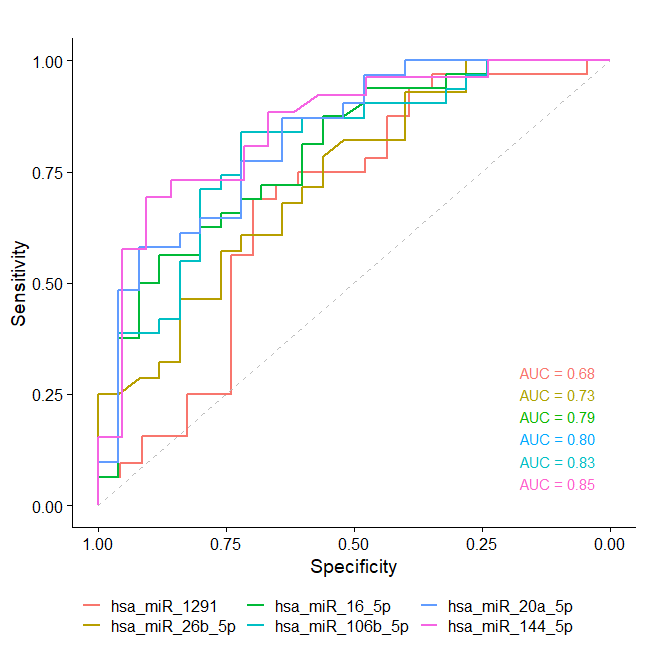
A B
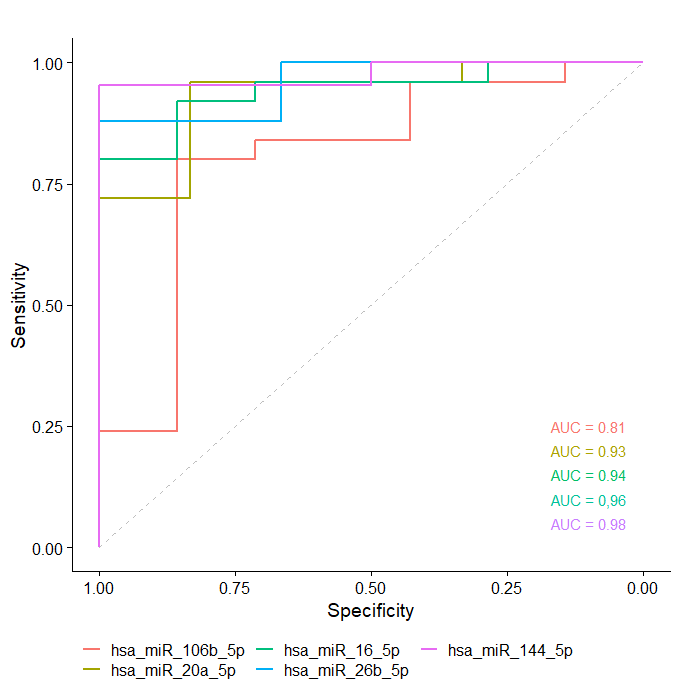


C


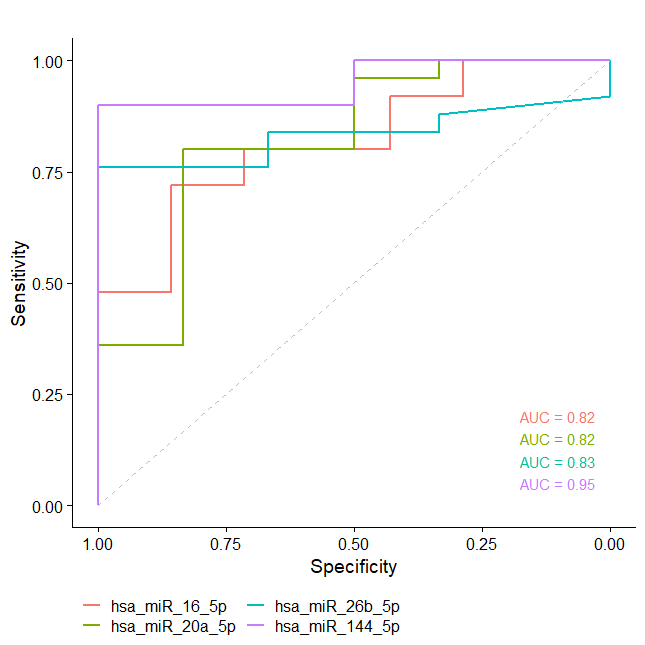


**Supplementary Figure 7.** ROC curves of studied miRNAs with statistically significant expression between the groups in female samples. Area Under the Curve (AUC) with at least 0.75 was considered as good ability in discriminating between comparations. A) ROC curve between non-LP and LP. B) ROC curve between non-LP and BL-LL pole. C) ROC curve between non-LP and BT-TT pole.

**Supplementary Table 4.** Potential miRNA biomarkers for male LP patients

| miRNA | Non-Lp vs Lp | p-value | AUC | Non-Lp vs BT-TT | p-value | AUC | Non-Lp vs BL-LL | p-value | AUC | BT-TT vs BL-LL | p-value | AUC |
| --- | --- | --- | --- | --- | --- | --- | --- | --- | --- | --- | --- | --- |
| *hsa-miR-1291* | ✔️ | 3.6e-05 | 0.85 | ✔️ | 3.7e-03 | 0.86 | ✔️ | 7.7e-04 | 0.85 | ✖️ | ns | - |
| *hsa-miR-20a-5p* | ✔️ | 1.9e-03 | 0.77 | ✖️ | ns | - | ✔️ | 0.03 | 0.78 | ✖️ | ns | - |
| *hsa-miR-144-5p* | ✔️ | 0.01 | 0.76 | ✖️ | ns | - | ✔️ | 4e-03 | 0.80 | ✖️ | ns | - |
| *hsa-miR-26b-5p* | ✖️ | 0,03 | 0.73 | ✖️ | ns | - | ✖️ | ns | - | ✖️ | ns | - |
| *hsa-miR-16-5p* | ✖️ | 0.03 | 0.72 | ✖️ | ns | - | ✖️ | ns | - | ✖️ | ns | - |
| *hsa-miR-106b-5p* | ✖️ | 0.032 | 0.69 | ✖️ | ns | - | ✖️ | ns | - | ✖️ | ns | - |
| *hsa-miR-126-5p* | ✖️ | ns | - | ✖️ | ns | - | ✖️ | ns | - | ✖️ | ns | - |
| *hsa-miR-15a-5p* | ✖️ | ns | - | ✖️ | ns | - | ✖️ | ns | - | ✖️ | ns | - |
| *hsa-let7f-5p* | ✖️ | ns | - | ✖️ | ns | - | ✖️ | ns | - | ✖️ | ns | - |

*ns: no significance

**Supplementary Table 5.** Potential miRNA biomarkers for female LP patients

| miRNA | Non-Lp vs Lp | | p-value | AUC | Non-Lp vs BT-TT | p-value | AUC | Non-Lp vs BL-LL | p-value | AUC | BT-TT vs BL-LL | p-value | AUC |
| --- | --- | --- | --- | --- | --- | --- | --- | --- | --- | --- | --- | --- | --- |
| *hsa-miR-144-5p* | | ✔️ | 1.8e-05 | 0.85 | ✔️ | 1.4e-03 | 0.81 | ✔️ | 4.3e-04 | 0.98 | ✔️ | 2.1e-03 | 0.95 |
| *hsa-miR-20a-5p* | | ✔️ | 3.3e-05 | 0.83 | ✔️ | 8.6-04 | 0.80 | ✔️ | 8.6e-04 | 0.93 | ✔️ | 0.02 | 0.82 |
| *hsa-miR-16-5p* | | ✔️ | 8.1e-05 | 0.79 | ✔️ | 3.6-03 | 0.75 | ✔️ | 1.7e-03 | 0.94 | ✔️ | 0.02 | 0.82 |
| *hsa-miR-106b-5p* | | ✔️ | 1.5e-04 | 0.80 | ✔️ | 1.2e-03 | 0.80 | ✔️ | 0.03 | 0.81 | ✖️ | ns | - |
| *hsa-miR-26b-5p* | | ✖️ | 1.9e-03 | 0.73 | ✖️ | 0.02 | 0.70 | ✖️ | 0.03 | 0.96 | ✔️ | 0.05 | 0.70 |
| *hsa-miR-1291* | | ✖️ | 0.048 | 0.68 | ✖️ | ns | - | ✖️ | ns | - | ✖️ | ns | - |
| *hsa-miR-15a-5p* | | ✖️ | ns | - | ✖️ | ns | - | ✖️ | ns | - | ✖️ | ns | - |
| *hsa-miR-126-5p* | | ✖️ | ns | - | ✖️ | ns | - | ✖️ | ns | - | ✖️ | ns | - |
| *hsa-let7f-5p* | | ✖️ | ns | - | ✖️ | ns | - | ✖️ | ns | - | ✖️ | ns | - |

*ns: no significance
